# Overexpressed MAGP1 Is Associated With a Poor Prognosis and Promotes Cell Migration and Invasion in Gastric Cancer

**DOI:** 10.3389/fonc.2019.01544

**Published:** 2020-01-17

**Authors:** Mengjie Wu, Yongfeng Ding, Xiaoxia Jiang, Yanyan Chen, Nan Wu, Linrong Li, Haiyong Wang, Yingying Huang, Nong Xu, Lisong Teng

**Affiliations:** ^1^Cancer Center, The First Affiliated Hospital, College of Medicine, Zhejiang University, Hangzhou, China; ^2^Department of Thoracic Surgery, First Affiliated Hospital of Zhengzhou University, Zhengzhou, China; ^3^Department of Otorhinolaryngology, The Second Affiliated Hospital, School of Medicine, Zhejiang University, Hangzhou, China

**Keywords:** gastric cancer, MAGP1, prognosis, biomarker, migration, invasion

## Abstract

Gastric cancer (GC) is a frequently occurring malignancy with high mortality rates. However, the underlying mechanism of GC progression is not very clear. The aim of this study is to reveal the inherent molecular mechanism and develop potential therapeutic targets for advanced GC. The microfibril-associated glycoprotein 1 (MAGP1), identified as a potential oncogene, was found upregulated in GC tissues and high MAGP1 expression was associated with aggressive clinicopathological features. Furthermore, the multivariate Cox regression analysis showed that high MAGP1 expression was an independent predictor of poor prognosis (HR = 2.37, 1.07–5.24; *P* = 0.033). Mechanistically, MAGP1 promoted the migration and invasiveness of GC cells. In addition, the genes co-expressed with MAGP1 were primarily enriched in focal adhesion and PI3K-Akt pathways. MAGP1 overexpression enhanced the phosphorylation of FAK, AKT, and mTOR, whereas its knockdown also inactivated these factors. Furthermore, the AKT inhibitor suppressed the phosphorylation of AKT, FAK, and mTOR in recMAGP1-treated AGS cells, as well as their migration and invasion capacities. Finally, correlation analysis indicated that MAGP1 is involved in AKT signaling in GC, and is clinically relevant. Taken together, MAGP1 is a promising prognostic marker and potential therapeutic target for advanced GC.

## Introduction

Gastric cancer (GC) is an aggressive gastrointestinal malignancy with high incidence and mortality rates worldwide, especially in Asia (1–3). GC patients are often diagnosed at the advanced stages due to lack of characteristic early symptoms, and frequent recurrence with distant metastasis is seen even after surgical resection, due to undetected micro-metastases (4, 5). For locally advanced GC, adjuvant or neoadjuvant therapy is usually implemented in combination with surgery. In metastatic GC, outcomes are poor, with median survival being around 1 year (6). Targeted therapy has improved the prognosis of various tumors, such as breast cancer, non-small cell lung cancer and colorectal cancer (7). In addition, novel molecular targeting agents that were effective in other malignancies have failed against GC. Therefore, it is essential to identify novel biomarkers and therapeutic targets for advanced GC.

Next-generation sequencing (NGS) has enabled the rapid and systematic analysis of large sets of tumor genomic data and helped elucidate the biological complexities of cancer cells, and identify potential therapeutic targets (8–10). Comparison of the gene-expression profiles of the metastatic (stage IV) and early stages (stage IA) can identify the differentially expressed genes (DEGs) and molecular signatures potentially correlated with tumor progression and metastasis in different cancer types, as well as novel biomarkers for cancer prognosis.

Microfibril associated glycoprotein 1 (MAGP1), a small extracellular matrix molecule (21 kDa), is coded by the *MFAP2* gene that is located on human chromosome 1p31 (11, 12). Its C-terminal end includes a matrix-binding domain (MBD) which tethers it to the extracellular matrix (ECM) (11, 12). Previous studies established MAGP1 as a protective factor in obesity and diabetes, which promoted thermogenesis by regulating the TGF-β/Smad3 signaling pathway (13). Loss of MAGP1 can affect the development of caudal blood vessels in zebrafish (14). Studies have also implicated MAGP1 in the progression of several cancers. For example, MAGP1 levels are higher in head and neck squamous cell carcinoma tissues, especially during metastatic growth, compared to that in adjacent normal tissues (15). In multiple myeloma, MAGP1 associated with the NF-kappaB/Snail/YY1/RKIP circuitry (16), and a MAGP2 homolog can promote metastasis of ovarian cancer (17). However, the expression pattern and function of MAGP1 in GC is not clear.

In this study, we identified MAGP1 as a potential oncogene in GC through transcriptomic analysis, and explored its expression levels, clinical relevance, and prognostic value in GC using both public databases and patient samples. Functional assays in GC cell lines further revealed the MAGP1-related signaling pathways. Our findings suggest that MAGP1 is an independent prognostic biomarker as well as a potential therapeutic target for advanced GC.

## Materials and Methods

### Tissue Samples and Cell Lines

A total of 143 GC and matched non-tumor tissue samples (ZJU cohort 1: *N* = 69 for qPCR; ZJU cohort 2: *N* = 74 for immunohistochemistry) were collected from patients referring to the Zhejiang University. The patients had been diagnosed with GC based on histopathological examination, and had not received adjuvant treatment before surgery. Tumor staging was determined according to the American Joint Committee on Cancer (seventh edition) criteria. This study was approved by the Ethics Committee of Zhejiang University College of Medicine, Zhejiang, China, and all patients provided informed written consent prior to sample collection.

Four human GC cell lines (HGC27, AGS, MKN45, andMGC803) were cultured in RPMI 1640 (Gibco, Rockville, MD, USA) supplemented with 10% fetal bovine serum (FBS; Gibco), the normal gastric cell line GES-1 was cultured in DMEM (Gibco, Rockville, MD, USA) supplemented with 10% fetal bovine serum (FBS; Gibco), and the SNU5 line was cultured in IMDM (Gibco, Rockville, MD, USA) supplemented with 20% FBS. All cell lines were purchased from the Shanghai Cell Bank of Chinese Academy of Sciences (Shanghai, China).

### RNA Extraction and Quantitative Real-Time PCR (RT-qPCR)

Total RNA was extracted from the tissue samples and cells using TRIzol reagent (Invitrogen, Carlsbad, California, USA) according to the manufacturer's instructions. One microgram RNA per sample was reverse transcribed to cDNA using the PrimeScript RT reagent kit (Takara, Kyoto, Japan), and the latter was amplified by qRT-PCR using the SYBR Green reaction system (Takara, Kyoto, Japan). The relative expression of MAGP1 was calculated by the 2-ΔΔCt method, with GAPDH as the internal control. The primer sequences were as follows: MAGP1 forward: 5′-CGCCGTGTGTACGTCATTAAC-3′ and reverse: 5′-CCATCACGCCACATTTGGA-3′, and GAPDH forward: 5′-GGAGCGAGATCCCTCCAAAAT-3′, and reverse: 5′-GGCTGTTGTCATACTTCTCATGG-3′.

### Immunohistochemistry (IHC)

Paraffin-embedded GC sections were deparaffinized in xylene, dehydrated through an ethanol gradient, and blocked with 3% H_2_O_2_ for 10 min. The slides were then heated in citrate buffer (pH 6) at 95°C for 15–20 min for antigen retrieval. The tissue sections were incubated overnight with anti-MAGP1 (1:200 diluted, Sigma, USA) and anti-P-AKT (1:100 diluted, Proteintech, USA) antibodies at 4°C, followed by a 30 min incubation with HRP-conjugated secondary antibody (ZSGB-bio, Beijing, China) at 37°C. After washing the slides thrice with PBS, the color was developed using DAB Chromogen (ZSGB-bio, Beijing, China). The slides were then rinsed with tap water and counterstained with hematoxylin. Five random fields per section were viewed under a light microscope, and the expression level of MAGP1 in the ECM of cancer cells was scored in terms of the intensity of staining and the percentage of positive-stained area. The positive rate was scored on a scale from 0 to 5 (0–no staining, 1– ≤ 20%, 2– ≤ 40%, 3– ≤ 60%, 4– ≤ 80%, and 5–>80%), and the staining intensity was graded as 0 (negative), 1 (weak), 2 (moderate), or 3 (strong).

### Small Interfering RNA (siRNA)-Mediated Knockdown

Negative control siRNA and two siRNAs targeting the human MAGP1 sequence were purchased from GenePharma company (Shanghai, China). Cells were transfected with Lipofectamine 3000 transfection reagent (Invitrogen, Carlsbad, California, USA) in Opti-MEM medium (Gibco, Carlsbad, California, USA). These two siRNAs sequences were: siMAGP1-1, sense, 5′-GGAGAUCUGUGUUCGUACATT-3′, antisense, 5′-UGUACGAACACAGAUCUCCTT-3′; si-MAGP1-2, sense, 5′-GUCCAGUACACCCACUAUATT-3′, antisense, 5′-UAUAGUGGGUGUACUGGACTT-3′.

### Cell Proliferation Assay

Cell proliferation was assessed using the Cell Counting Kit-8 (CCK-8) (Dojindo Laboratories, Kumamoto, Japan) according to the manufacturer's instructions. Briefly, 3,000 cells were seeded per well of a 96-wells plate in 100 μl medium, followed by 10 μl CCK-8 reagent in 100 μl complete medium after 0, 24, 48, and 72 h post-transfection. After incubating the cells for another 2 h, the absorbance at 450 nm was measured.

### Cell Migration and Invasion Assays

The cell migration and invasion assays were performed using 24-well format Transwell chambers with 8.0-um pore size polycarbonate filter inserts (Millipore, Washington, DC, USA). A total of 5 x 10^4^ cells were seeded in the uncoated upper chamber per well for the cell migration assays, while 1 x 10^5^ cells were seeded in Matrigel (BD Biosciences, Lake Franklin, NJ, USA)-coated chambers for the cell invasion assays. The lower chambers were filled with 900 μl complete medium. After 24 h incubation at 37°C, the cells that had migrated from the upper to the lower surface of the filters were washed thrice with PBS, fixed in 95% ethanol for 10 min, stained with 0.05% crystal violet for 10 min at the room temperature, and washed twice with PBS. The number of cells were counted in at least five random fields (100x) per sample under a light microscope.

### Western Blotting

Cells were lysed using the radio-immunoprecipitation assay (RIPA) lysis buffer (Beyotime, Shanghai, China) supplemented with phenylmethanesulfonyl fluoride (PMSF) (Bioship, Anhui, China) and a protease inhibitor cocktail (Sigma, USA). The proteins were quantified by the BCA Protein Assay Kit (Thermo Fisher Scientific, Massachusetts, USA), and equal amounts per sample were separated by SDS-PAGE and transferred to a PVDF membrane. The membranes were blocked with 10% skim milk for 1 h at room temperature, and then incubated overnight with primary antibodies against MFAP2, p-AKT, AKT, p-FAK, FAK, p-mTOR, mTOR (1:1,000, Abcam, Cambridge, UK) and GAPDH (Cell signal, Massachusetts, USA) at 4°C. After incubating for 1 h with an HRP-conjugated secondary antibody (Cell signaling, Massachusetts, USA) at room temperature, the membranes were washed thrice with TBST, and the positive bands were visualized using an enhanced chemiluminescence (ECL) reagent (Bio-red, California, USA).

### Bioinformatics Analyses

The RNA-seq gene expression data (RNAseqV2 RSEM) of GC and the corresponding clinicopathological information was download from The Cancer Genome Atlas (TCGA) via cBioPortal (https://www.cbioportal.org/). The DESeq package (18) was used to screen for differentially expressed genes (DEGs) from 18,734 genes between the metastatic and early stages, with false discovery rate (FDR)-adjusted *p* < 0.05 and absolute fold change >2 as the thresholds. The prognostic value of DEGs in GC was further evaluated using the Kaplan-Meier plotter (http://kmplot.com/analysis/) and GEPIA (http://gepia.cancer-pku.cn/detail.php) database. The patient samples were divided into two cohorts according to the median expression of MAGP1 (high vs. low expression), and the overall survival (OS) and progression-free survival (PFS) were analyzed. Log rank *p*-value and hazard ratio (HR) with 95% confidence intervals were calculated. The genes with significant prognostic value in both databases were identified as the survival-associated genes. In addition, the intrinsic mechanisms of these genes in GC were determined by extracting the co-expressed genes using cBioPortal (http://www.cbioportal.org) and Cancer Genomics and Coexpedia (http://www.coexpedia.org), with |Spearman's r| ≥ 0.4 as the threshold. Finally, gene ontology (GO) and Kyoto Encyclopedia of Genes and Genomes (KEGG) pathway analyses were performed in David v6.7 (http://david.abcc.ncifcrf.gov/) to elucidate the enrichment of these genes in biological processes and signaling pathways.

### Statistical Analysis

Two or multiple groups were compared using *t*-test or one way ANOVA, respectively. The chi-square and Yates' continuity corrected chi-square tests were used to analyze the significance of categorical data. Survival rates were calculated by the Kaplan–Meier method and compared using the log-rank test. The univariate and multivariate analyses were performed using the Cox regression model. Variables with *P* < 0.05 were included in the multivariate analysis to identify the independent prognostic factors. Statistical analysis was performed using GraphPad Prism 8 software. *P* < 0.05 was considered statistically significant.

## Results

### MAGP1 Is a Potential Oncogene in GC

**Figure 1:**
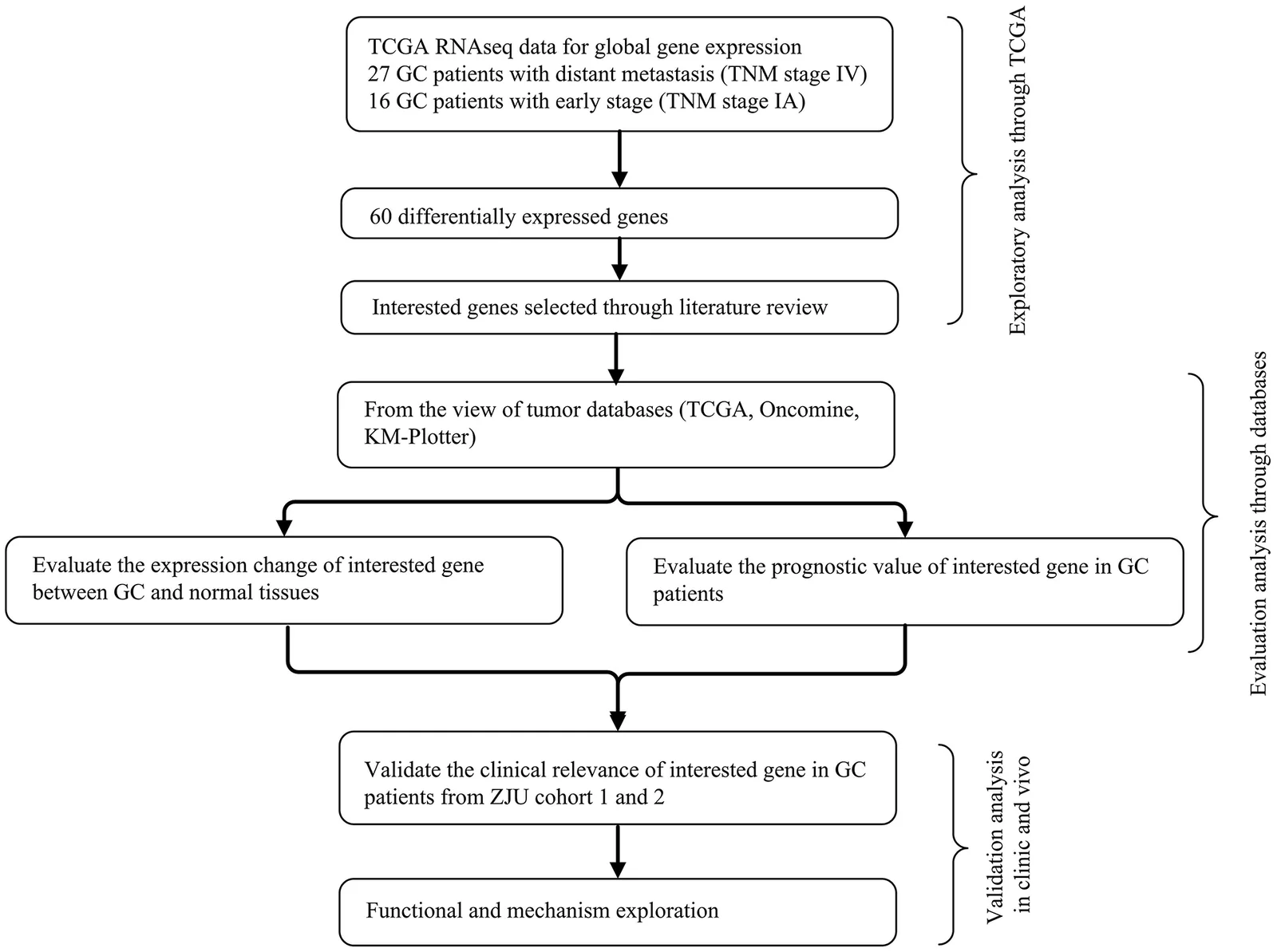
The integrative analytic strategy in this study. GC, Gastric cancer; KM–Plotter, Kaplan-Meier Plotter database; ZJU, Zhejiang University.

To identify and validate the key genes involved in GC metastasis, we developed a multi-step analytical strategy (Figure 1). The three steps were as follows: (1) identifying the putative DEGs involved in GC metastasis through TCGA; (2) determining the association between the DEGs and the clinical prognosis of GC using TCGA, Oncomine and KM-Plotter; (3) validation of the DEGs by analyzing their expression levels in two independent cohorts (ZJU cohort 1 and ZJU cohort 2), and by functional assays. The GC samples retrieved from TCGA were divided into the metastatic [TNM (tumor-node-metastasis) stage IV, *N* = 27) and early stage (TNM stage IA, *N* = 16) groups, in order to identify the putative DEGs involved in GC metastasis. Sixty progression-associated DEGs were identified using the DESeq R package, of which only four genes (FBLN5, GGT5, MAGP1, and PRICKLE1) were ascertained as survival-associated genes based on Kaplan-Meier plotter database and GEPIA database. Based on our literature review, we selected MAGP1 for the subsequent experiments since few studies had explored the relationship between MAGP1 and cancer (details in Table S1).

### MAGP1 Is Upregulated in GC

**Figure 2:**
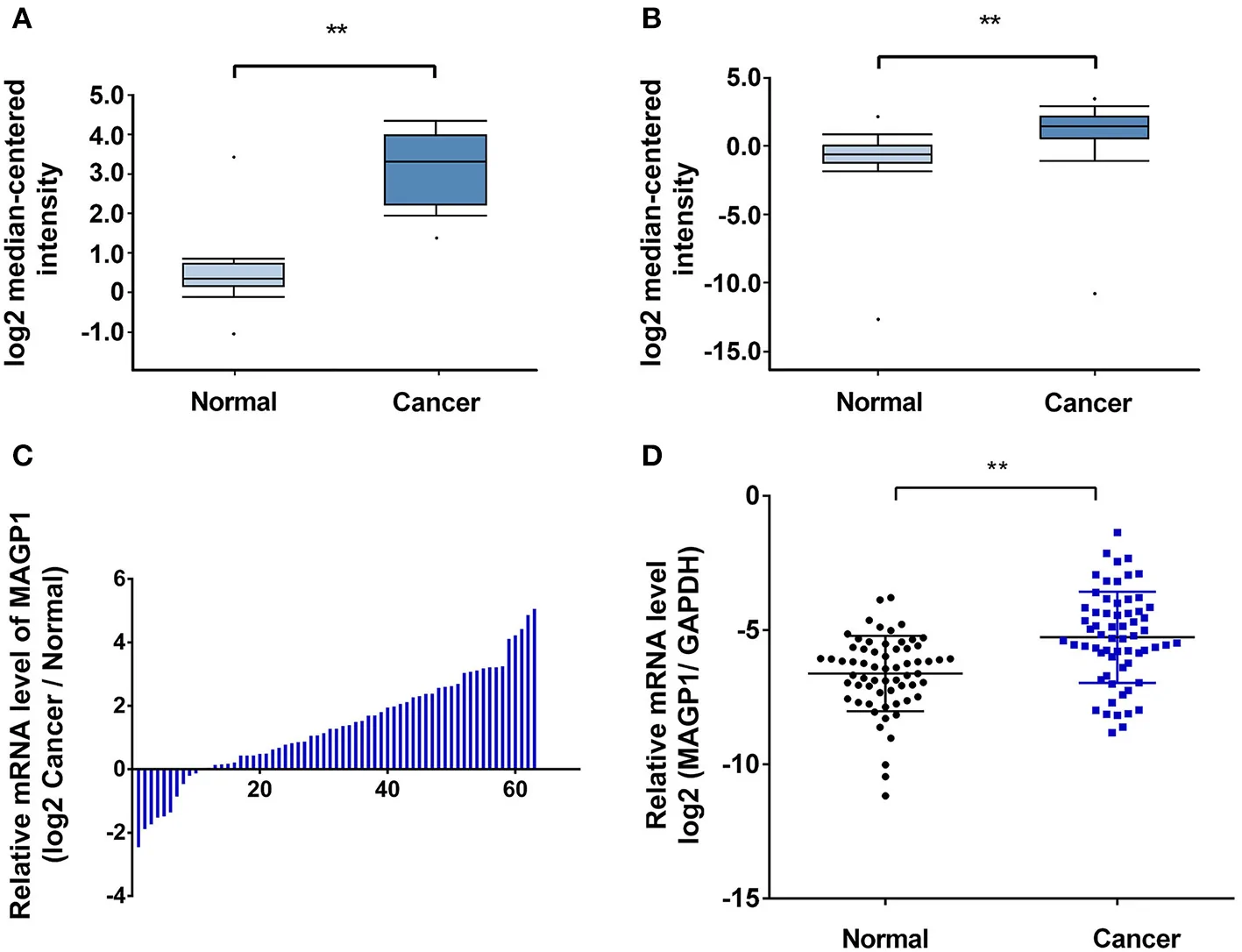
High mRNA expression of *MAGP1* in gastric cancer (GC). **(A,B)** *MAGP1* mRNA levels of GC vs. normal gastric tissue in Oncomine database (Wang Gastric, Cui Gastric). **(C,D)** *MAGP1* mRNA levels of GC vs. normal gastric tissue in ZJU cohort 1. Relative expression of *MAGP1* mRNA in gastric cancer tissues and their corresponding normal tissues was determined by qRT-PCR and expressed as –ΔΔCT (**P < 0.01).

**Figure 3:**
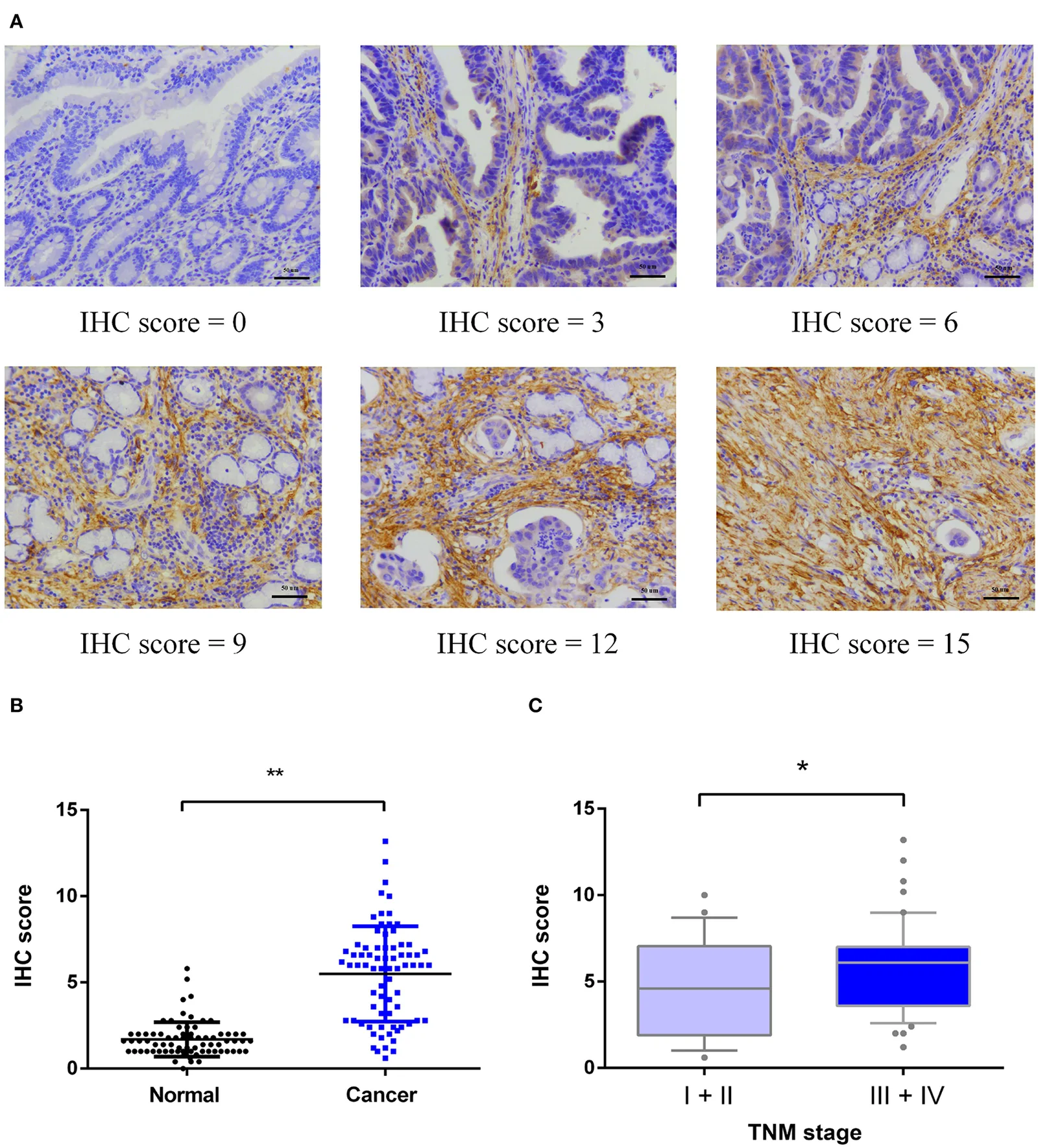
Immunohistochemical (IHC) analysis of MAGP1 protein expression in ZJU cohort 2. **(A)** Representative immunohistochemical staining of MAGP1 in GC tissues and adjacent normal tissues (Original magnification x200, scale bar = 50 um). **(B)** Expression comparison between GC tissues and matched normal tissues by immunohistochemistry. **(C)** Expression comparison of GC with different TNM stages (I+II, N = 24; III+IV, N = 50) (*P < 0.05; **P < 0.01).

As shown in Figures 2A,B, MAGP1 was significantly upregulated in the GC samples compared to the corresponding normal gastric tissues in the Oncomine database (https://www.oncomine.org). To validate the overexpression of MAGP1 in GC, the mRNA levels 63 paired frozen GC and normal gastric tissues were analyzed (ZJU cohort 1), and MAGP1 mRNA levels were significantly higher in the tumor tissues (*P* < 0.05, Figures 2C,D). In addition, the intestinal, diffuse and mixed histological types of GC (different Lauren's histological types), each had higher MAGP1 expression than the normal gastric tissues (Figure S1). The *in situ* MAGP1 protein levels were also analyzed in 74 paired GC and corresponding normal tissues (ZJU cohort 2) (Figure 3A), and was significantly upregulated in the GC samples (Figure 3B). Furthermore, MAGP1 expression was higher in the stage III and IV samples compared to that of stage I and II (*P* < 0.05, Figure 3C). Taken together, MAGP1 is overexpressed in GC, especially in patients with advanced stages.

### MAGP1 Overexpression Is Associated With Poor Prognosis of GC Patients

**Figure 4:**
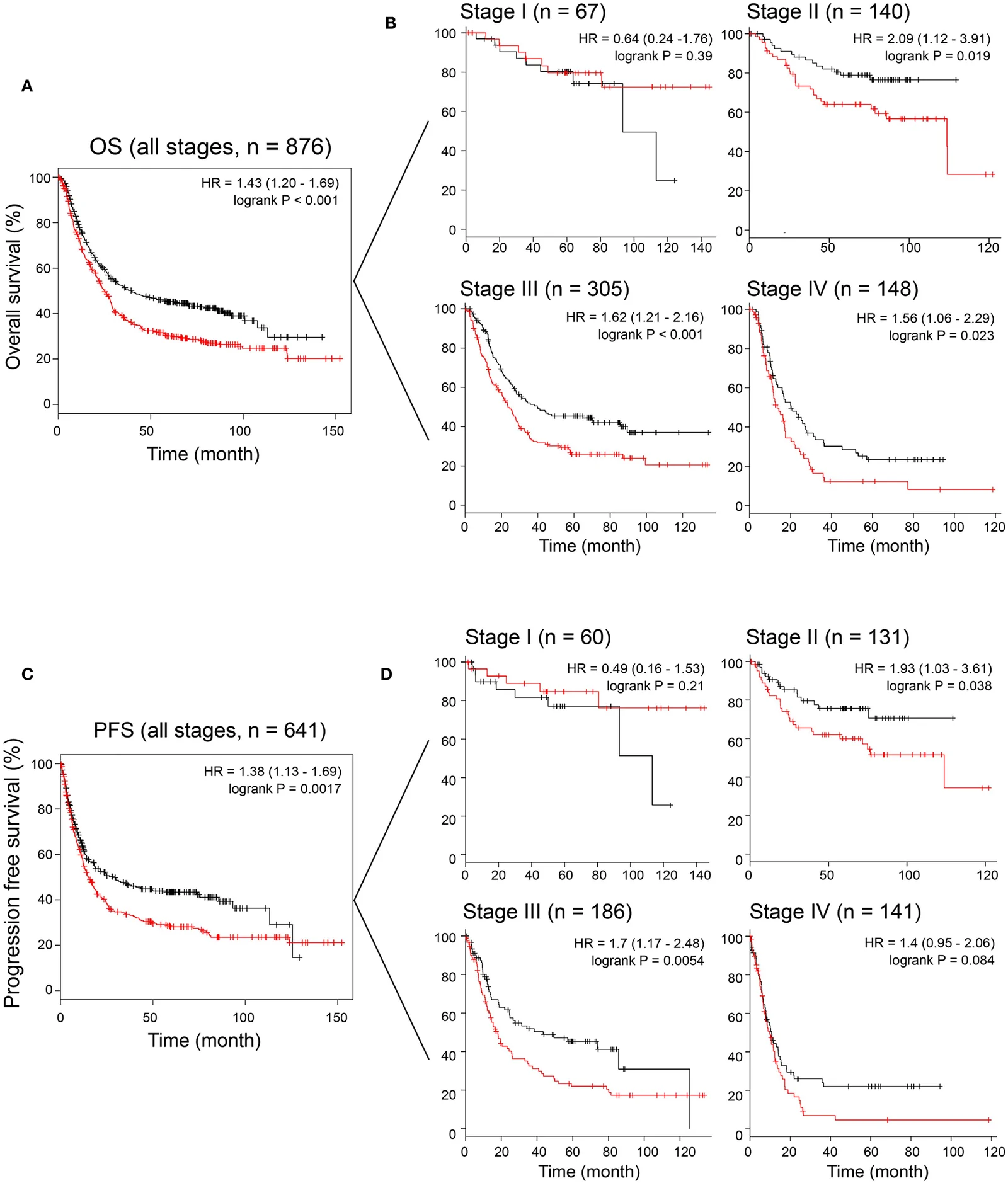
The association between *MAGP1* mRNA expression and prognosis of gastric cancer using K–M plotter data with the Affy ID 203417_at. **(A)** Overall survival and **(B)** disease free survival were showed between high (red) and low (black) expression group using K–M plotter data. **(C)** Overall survival was showed between high (red) and low (black) expression group in stage I–IV. **(D)** Progression free survival was showed between high (red) and low (black) expression group in stage I–IV.

To assess the prognostic value of MAGP1 expression in GC patients, we conducted survival analysis using KM-plotter database. As shown in Figures 4A,C, GC patients with overexpressing MAGP1 had remarkably shorter OS (*P* = 0.00004) and PFS (P = 0.002), compared to those with low MAGP1 mRNA levels. Further stratification according to the TNM stages showed that high levels of MAGP1 were significantly associated with poor OS in the TNM stage II (*P* = 0.019), III (*P* = 0.00092), and IV (*P* = 0.023) patients (Figure 4B). Similarly, GC patients with TNM stage II or III with high MAGP1 levels had significantly shorter PFS than those with low MAGP1 levels (Figure 4D). In addition, we also found that MAGP1 overexpression was significantly associated with poor prognosis (both OS and PFS) in GC patients with different Lauren classifications (Figure S2).

**Figure 5:**
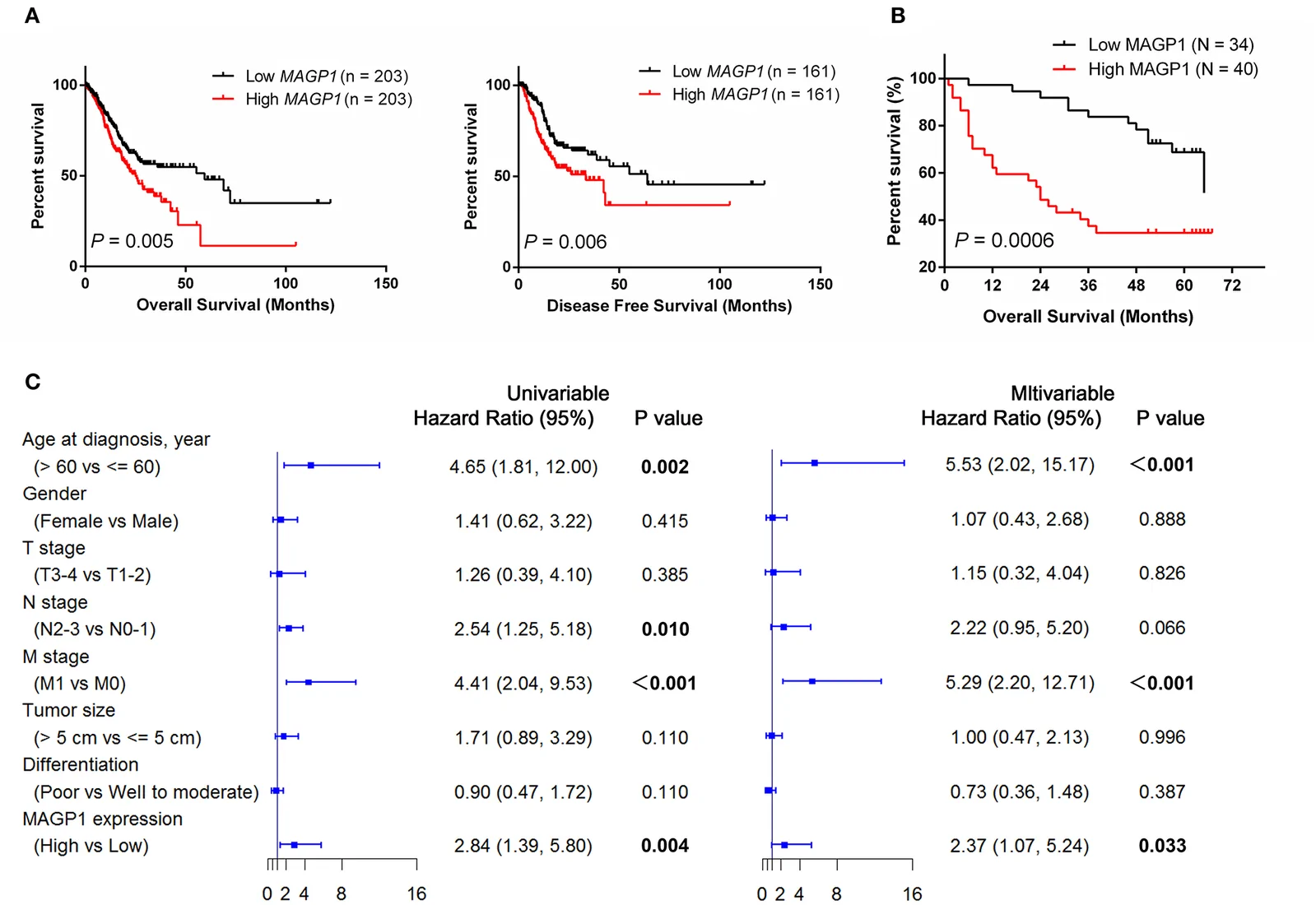
The association between MAGP1 expression and prognosis of patients with gastric cancer. **(A)** Overall survival and **(B)** Progression free survival were showed between high and low expression group of *MAGP1* mRNA using TCGA data. **(C)** The association between MAGP1 expression and prognosis of patients with gastric cancer in ZJU cohort 2.

We also performed the survival analysis using the GC data of TGCA, and found that high MAGP1 mRNA expression predicted worse prognosis (Figure 5A). In order to validate the prognostic value of MAGP1 at the protein level, we collected and follow-up data and clinicopathological features for ZJU cohort 2 (Table 1). We found that the OS rate in the high MAGP1 protein expression group was significantly lower than that in low expression group (*P* = 0.0006, Figure 5B). High MAGP1 expression was significantly associated with lymph node metastasis, tends to develop more distant metastasis and have poorer differentiation (details in Table S2). In addition, univariate Cox regression analysis showed that age at diagnosis (> 60 vs. < = 60, HR = 4.65), N stage (N2-N3 vs. N0-N1), M stage (M1 vs. M0), and MAGP1 expression (High vs. Low) were associated with poor OS, and multivariate analysis further established MAGP1 expression as an independent prognostic factor for OS (*P* = 0.033, Figure 5C).

**Table 1:** The clinicopathological features of GC in ZJU cohort 2.

**Clinicopathological features**	**No. patients (%) (*N* = 74)**
**Gender**	
Male	57 (77)
Female	17 (23)
**Age**	
≤60	27 (36)
>60	47 (64)
**AJCC stage**	
I–II	16 (22)
III–IV	58 (78)
**T stage**	
T1-2	7 (9)
T3-4	67 (91)
**N stage**	
N0-1	33 (45)
N2-3	41 (55)
**M stage**	
M0	62 (84)
M1	12 (16)
**Tumor size**	
≤5 cm	45 (61)
>5 cm	29 (39)
**Differentiation**	
Well to moderate	37 (50)
Poor	37 (50)
**Borrmann classification**	
I	9 (12.5)
II–III	56 (77.8)
IV	7 (9.7)

### MAGP1 Regulates Migration and Invasion of Human GC Cell Lines

**Figure 6:**
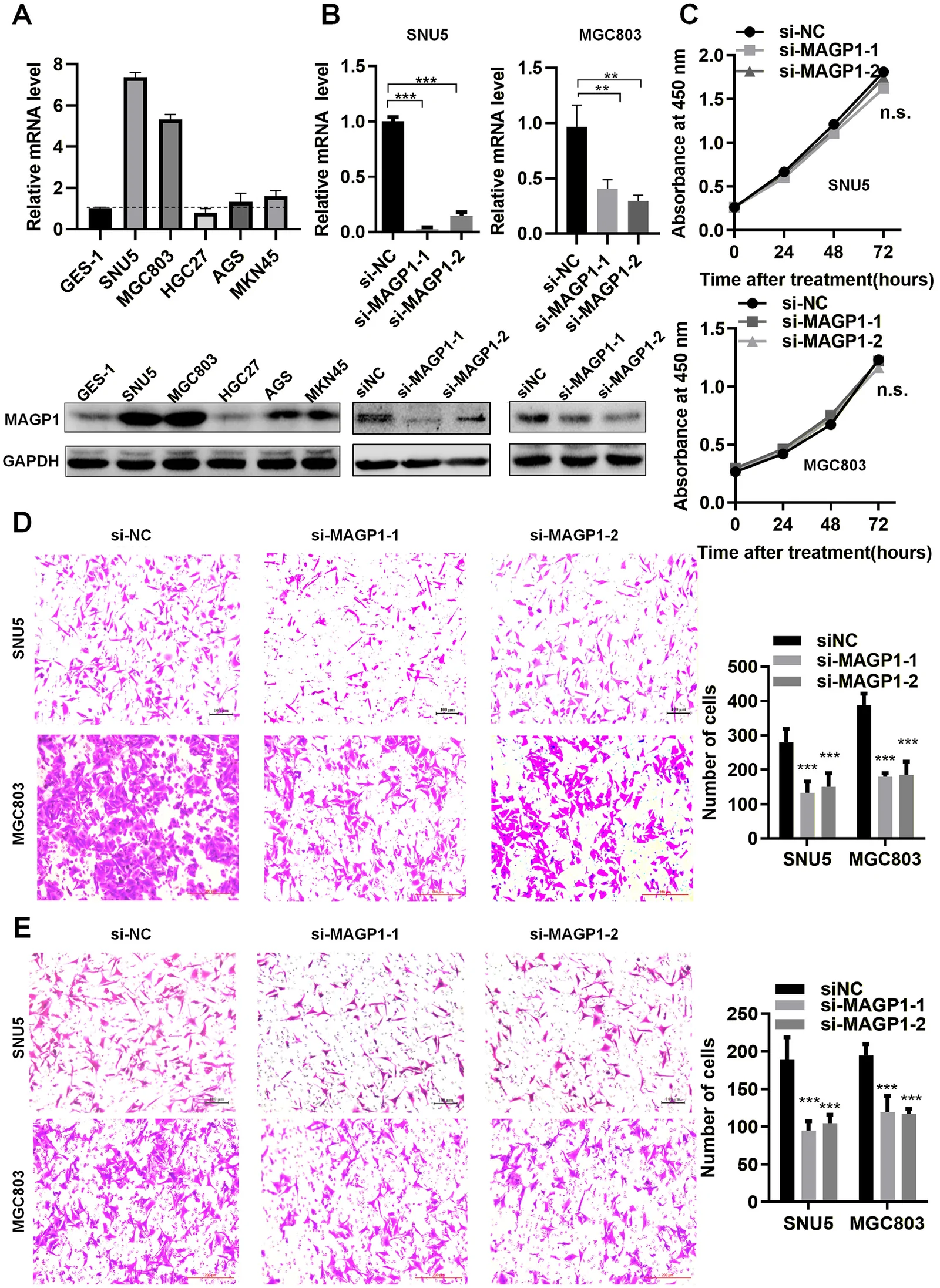
MAGP1 regulates migration and invasion of human GC cell lines. **(A)** mRNA and protein expression levels of MAGP1 were checked in a panel of five human GC cell lines and normal gastric cells (GES-1) using RT-qPCR and immunoblotting. **(B)** mRNA and protein expression level of MAGP1 were efficiently inhibited by si-MAGP1-1 and si-MAGP1-2 in SNU5 and MGC803 cells. **(C)** CCK-8 assay, **(D)** migration assay, and **(E)** invasion assay were performed in SNU5 and MGC803 cells transfected with si-MAGP1-1, si-MAGP1–2, and control siRNA (**P < 0.01; ***P < 0.001).

**Figure 7:**
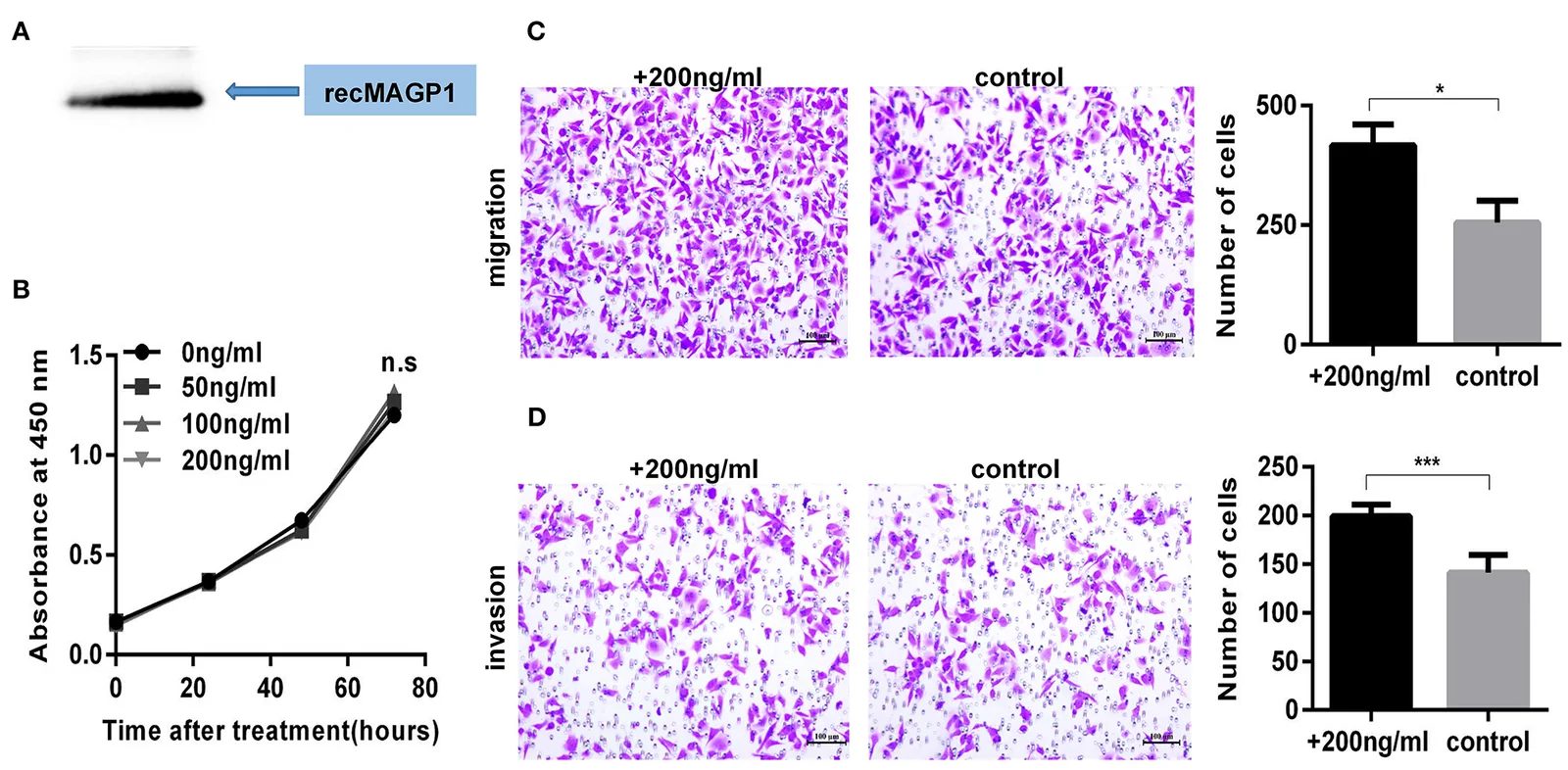
MAGP1 Stimulates migration and invasion of AGS cell line. **(A)** Recombinant MAGP1 was checked by immunoblotting. **(B)** CCK8 assay, **(C)** migration assay, and **(D)** invasion assay of AGS cells was performed in the presence of recombinant MAGP1 (*P < 0.05; ***P < 0.001).

The MAGP1 mRNA and protein levels of five GC cell lines (SNU5, MGC803, HGC27, AGS, MKN45) and normal gastric cell line (GES-1) were measured, and found that MAGP1 expressed higher in SNU5 and MGC803, and lower in normal gastric cell line (GES-1) compared to the most of cell lines, including SNU5, MGC803, AGS, MKN45 (Figure 6A). To determine the biological function of MAGP1 in GC, we knocked down MAGP1 expression in the SNU5 and MGC803 cells using siRNA, and examined the proliferation, migration and invasion abilities of the cells (Figure 6B). MAGP1 knockdown significantly inhibited the migration and invasion of SNU5 and MGC803 cells compared to the control cells, but no significant difference was seen between the proliferation rate of the wild-type and si-MAGP1-1/si-MAGP1-2 transfected cells (Figures 6C–E). In addition, we analyzed the effect of MAGP1 overexpression on the motility of the low-expressing AGS cells (Figure 7A). Treatment with 200 ng/ml recMAGP1 protein (USCN Business company, Wuhan, China) for 72 h had no effect on the proliferation of AGS cells, but significantly enhanced their migration and invasiveness compared to the untreated cells (Figures 7B–D).

### MAGP1 Is Involved in Focal Adhesion and PI3K-AKT Signaling Pathways in GC

**Figure 8:**
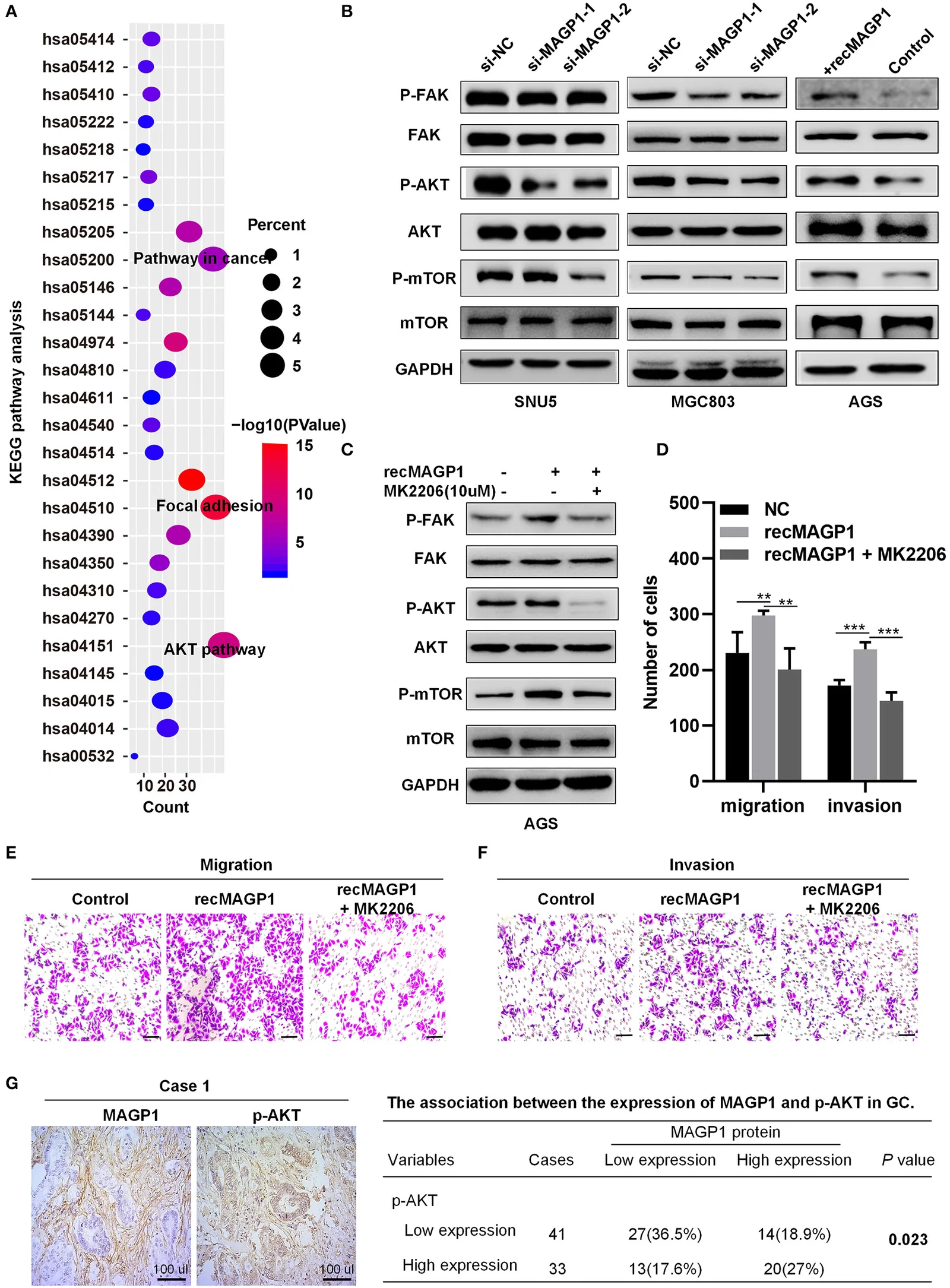
MAGP1 is involved in Focal adhesion and PI3K-AKT signaling pathways in GC. **(A)** KEGG pathway. KEGG, Kyoto Encyclopedia of Genes and Genomes. **(B)** The protein expression levels of *p*-FAK, FAK, *p*-AKT, AKT, *p*-mTOR, and mTOR in GC cell lines after MAGP1 silencing or the presence of recombinant MAGP1. **(C)** The protein expression levels of *p*-FAK, FAK, *p*-AKT, AKT, *p*-mTOR, and mTOR in AGS cell after treating with recMAGP1 and MK2206. **(D–F)** Migration and invasion assay of AGS cells treated with recMAGP1 and MK2206. **(G)** Representative IHC images and clinical relevance of *p*-AKT and MAGP1 (SP, x200). (**P < 0.01; ***P < 0.01).

Seven hundred and forty-five genes co-expressed with MAGP1 in GC were identified by the union cluster in cBioPortal (http://www.cbioportal.org) for cancer genomics (|Spearman's r| ≥ 0.4) and Coexpedia (http://www.coexpedia.org) (details in Table S3). The significant GO terms are shown in Figure S3, and the enriched KEGG pathways were focal adhesion and PI3K-AKT signaling (Figure 8A) (details in Tables S4, S5). Furthermore, the Western blot was performed and the results showed that the total expression of AKT, FAK, and mTOR were not changed, but the expression levels of p-AKT, p-FAK, and p-mTOR were significantly downregulated in si-MAGP1-1 /si-MAGP1-2 transfected GC cells. In addition, the expression of p-AKT, p-FAK and p-mTOR were upregulated when treated with 200 ng/ml recMAGP1 in AGS (Figure 8B). Meanwhile, the AKT inhibitor (MK2206, Selleck, USA) suppressed the expression of p-AKT, p-FAK, and p-mTOR in recMAGP1-treated AGS cells (Figure 8C). Inhibition of AKT also reversed the increased migration and invasiveness of the recMAGP1-treated cells (Figures 8D–F). Finally, MAGP1 levels correlated positively with that of p-AKT (*P* = 0.023, Figure 8G) in 74 GC specimens, indicating that MAGP1 is clinically relevant and involved in focal adhesion and PI3K-AKT signaling pathways.

## Discussion

In this study, we conducted a progression pattern-specific transcriptome analysis and identified four genes as candidate biomarkers associated with progression and prognosis of GC. Microfibril associated glycoprotein 1 (MAGP1), as one of the survival-associated genes, was selected for further analysis. We found that MAGP1 expression was upregulated in GC tissues and correlated with lymph node metastasis and poor prognosis. Mechanistically, MAGP1 is involved in focal adhesion and PI3K-AKT signaling pathways in GC cells, and is clinically relevant. These results suggest that MAGP1 is a good biomarker for prognosis of GC patients and can be used as potential therapeutic target for advanced GC.

Up to now, advances in the high-throughput sequencing techniques have generated large amounts of data that have been used to identify key molecules for risk stratification, prognosis prediction and therapeutic targets development in various types of cancers (19, 20). For instance, Cheong et al. (21) identified and validated predictive biomarkers of chemotherapy response in patients with resectable GC through transcriptome datasets analysis. By analyzing the gene expression profile of metastatic and non-metastatic nasopharyngeal carcinoma tissues, Tang et al. (22) identified a gene-expression signature predictive of distant metastasis in patients with loco-regionally advanced cancer. Tumor-node-metastasis (TNM) stage, which is the major determinant of appropriate treatment and prognosis, is an anatomically based system that records the primary and regional nodal extent of the tumor and the absence or presence of metastases. The survival time of advanced GC patients with distant metastasis in stage IV (Any T; any N; M1) are shorter than that of early GC patients without lymph node metastasis, distant metastasis in stage IA (T1; N0; M0) (23). In the present study, we identified 60 potential biomarkers associated with GC progression through transcriptome analysis between the metastatic (stage IV) and early stages (stage IA) and literature review. Several genes have been previously associated with tumor progression, including that of GC. For example, Integrin subunit beta 1 (ITGB1) regulates multiple pathophysiological signaling pathways and promotes metastasis of gastric, prostate, breast, and head and neck squamous cell carcinoma (HNSCC) cells (24, 25). Furthermore, the metalloprotease inhibitor 3 encoding gene (TIMP3) correlates with metastasis and poor prognosis in gastric, colorectal, breast, brain, HNSCC, and bladder carcinoma (26). In addition, we furtherly extracted four survival-associated genes using Kaplan-Meier plotter and GEPIA databases.

In this study, we focused on MAGP1 since few studies had explored the relationship between MAGP1 and cancer, except the role in obesity, thermogenesis and homeostasis (12, 27). We found that MAGP1 was overexpressed in most digestive system tumors like gastric cancer, cholangiocarcinoma, esophageal cancer, colon cancer, and rectal adenocarcinoma. We found that MAGP1 was overexpressed in most digestive system tumors like gastric cancer, cholangiocarcinoma, esophageal cancer, colon cancer, and rectal adenocarcinoma (Figure S4). We subsequently found that MAGP1 mRNA levels were higher in all Lauren classifications of GC and correlated with lymph node metastasis. Silveira et al. (15) reported MAGP1 expression were found upregulated in HNSCC tissue, especially in lymph node metastasis, compared to adjacent normal tissues. We found that high MAGP1 expression was significantly associated with poor prognosis in GC patients regardless of the TNM stages or Lauren classifications, and was an independent risk factor. Thus, our results indicated that MAGP1 might play an important role in the progression of GC.

To explore the biological roles of MAGP1 involved in GC, we conducted functional assays in multiple human GC cell lines. MAGP1 knockdown inhibited the migration and invasion of GC cells, but had no effect on their proliferative capacities, while treatment of GC cells with recMAGP1 had the opposite effects. In addition, GO analysis indicated that MAGP1 involved in positive regulation of cell migration. Taken together, MAGP1 is associated with aggressive tumor behavior via increased migration and invasion. In a previous report, MAGP2, as the homologous protein of MAGP1, were found associated with metastatic potential of ovarian cancer (28). KEGG pathway analysis of MAGP1 co-expressed genes further showed an enrichment of focal adhesion, PI3K-AKT signaling and TGF-β signaling. Previous studies showed MAGP1 was involved in several phenotypic abnormalities of the ECM by regulating TGF-β activity, not impact structural features of ECM (29). And Fibrillin-1 can indirectly mediate TGF-β pathway by binding MAGP1, which tethered the active form of TGF-β to the microfibril (30). Meanwhile, ECM was reported to play an important role in metastasis of GC (31). The interaction between MAGP1 and ECM indicates MAGP1 may contribute to migration and invasion of GC cells. Interestingly, one recent study has shown that MAGP1 might promote EMT in GC cells by activating TGF-β/SMAD2/3 signaling pathway (32). Focal adhesion kinase (FAK), as a critical role of the focal adhesion pathway, can mediate many cellular metabolic processed, including cell growth, metastasis, survival, and closely associated with the development of cancers (33). Moreover, PI3K-AKT signaling pathway regulates basic molecular feature of cancer including cell survival, proliferation, migration, and invasion of cancer cells in many tumors, including GC (34, 35). AKT and mTOR act as crucial makers and play important roles in the PI3K-AKT signaling pathway (35). In our study, silencing MAGP1 inhibited the activity of FAK, while the phosphorylation of FAK was upregulated after treating with recMAGP1 in GC cells. And we observed that inhibiting MAGP1 in GC cells could also inhibit the activation of AKT and mTOR. By contrast, recMAGP1 protein promoted FAK, AKT, mTOR phosphorylation in GC cells. An AKT inhibitor abrogated the MAGP1-mediated upregulation of p-FAK and p-mTOR, as well as migration and invasiveness of GC cells. Finally, the amount of p-AKT correlated positively with MAGP1 levels. Taken together, MAGP1 is involved in focal adhesion and PI3K-AKT signaling pathways in GC.

In conclusion, high levels of MAGP1 in GC tissues correlate with poor prognosis of the patients. MAGP1 is a potential onco-protein that promotes GC cell migration and invasion in GC cells. In additional, MAGP1 is involved in focal adhesion and PI3K-AKT signaling pathways in GC cells, and is clinically relevant. Therefore, this study indicates MAGP1 is a promising prognostic biomarker and a potential therapeutic target for GC.

## Data Availability Statement

The datasets analyzed for this study can be found in cBioPortal (https://www.cbioportal.org/) and Oncomine (https://www.oncomine.org).

## Ethics Statement

This study was approved by the Ethics Committee of Zhejiang University College of Medicine, Zhejiang, China. Samples were collected from patients referring to the Zhejiang University, who provided written informed consent.

## Author Contributions

LT, MW, and YD conceived and designed the study. MW and XJ performed the cell line studies. MW, YD, YC, and NW wrote the manuscript and assessed the IHC score. LL, HW, YH, and NX reviewed the clinical records and conducted the statistical analysis. All authors read and approved the final manuscript.

### Conflict of Interest

The authors declare that the research was conducted in the absence of any commercial or financial relationships that could be construed as a potential conflict of interest.

## Supplementary Material

Table S1 The summary of 60 putative biomarkers.

Table S2 Association between MAGP1 expression and clinicopathological features of GC.

Table S3 745 genes co-expressed with MAGP1 in GC.

Table S4 GO analysis of MAGP1 co-expressed genes in GC.

Table S5 KEGG pathway analysis of MAGP1 co-expressed genes in GC.

Figure S1 MAGP1 mRNA expression in different gastric Lauren classifications. Analysis of MAGP1 mRNA expression in Chen et al. **(A–C)**, Cho et al. **(D–F)**, and Derrico et al. **(G–I)** gastric datasets. Boxes, 25th−75th percentile; whisker, 10th and 90th percentile; points, minimum and maximum. All data was achieved from Oncomine database.

Figure S2 The association between mfap2 mRNA expression and prognosis of gastric cancer with different Lauren classifications using K–M plotter data. **(A)** Overall survival was showed between high and low expression group of mfap2 in intestinal, diffuse, and mixed Lauren classification. **(B)** Progress survival was showed between high and low expression group of mfap2 in intestinal, diffuse, and mixed Lauren classification.

Figure S3 GO analysis of MAGP1 co-expressed genes in GC. **(A)** Biological processes (BPs); **(B)** Cellular components (CCs); **(C)** Molecular factors (MFs). GO, gene ontology.

Figure S4 The MAGP1 mRNA expression in digestive system tumors using Firehose. Red color represented tumors and blue color represented corresponding normal tissues. RSEM, RNASeq by expectation maximization. CHOL, cholangiocarcinoma; COAD, colon adenocarcinoma; ESCA, esophageal carcinoma; LIHC, liver hepatocellular carcinoma; PAAD, pancreatic adenocarcinoma; READ, rectum adenocarcinoma; STAD, stomach adenocarcinoma.
