# Correction: Overexpressed MAGP1 is associated with a poor prognosis and promotes cell migration and invasion in gastric cancer

**DOI:** 10.3389/fonc.2026.1979409

**Published:** 2026-10-23

**Authors:** Mengjie Wu, Yongfeng Ding, Xiaoxia Jiang, Yanyan Chen, Nan Wu, Linrong Li, Haiyong Wang, Yingying Huang, Nong Xu, Lisong Teng

**Affiliations:** 1 Cancer Center, The First Affiliated Hospital, College of Medicine, Zhejiang University, Hangzhou, China; 2 Department of Thoracic Surgery, First Affiliated Hospital of Zhengzhou University, Zhengzhou, China; 3 Department of Otorhinolaryngology, The Second Affiliated Hospital, School of Medicine, Zhejiang University, Hangzhou, China

**Keywords:** gastric cancer, MAGP1, prognosis, biomarker, migration, invasion

There was a mistake in **Figure 6** as published. The images assigned to the “SNU5 Migration si-MAGP1-1” and “MGC803 Migration si-MAGP1-2” groups within **Figure 6D** were erroneously chosen while assembling the composite **Figure 6**. The corrected **Figure 6** appears below.

**Figure 6:**
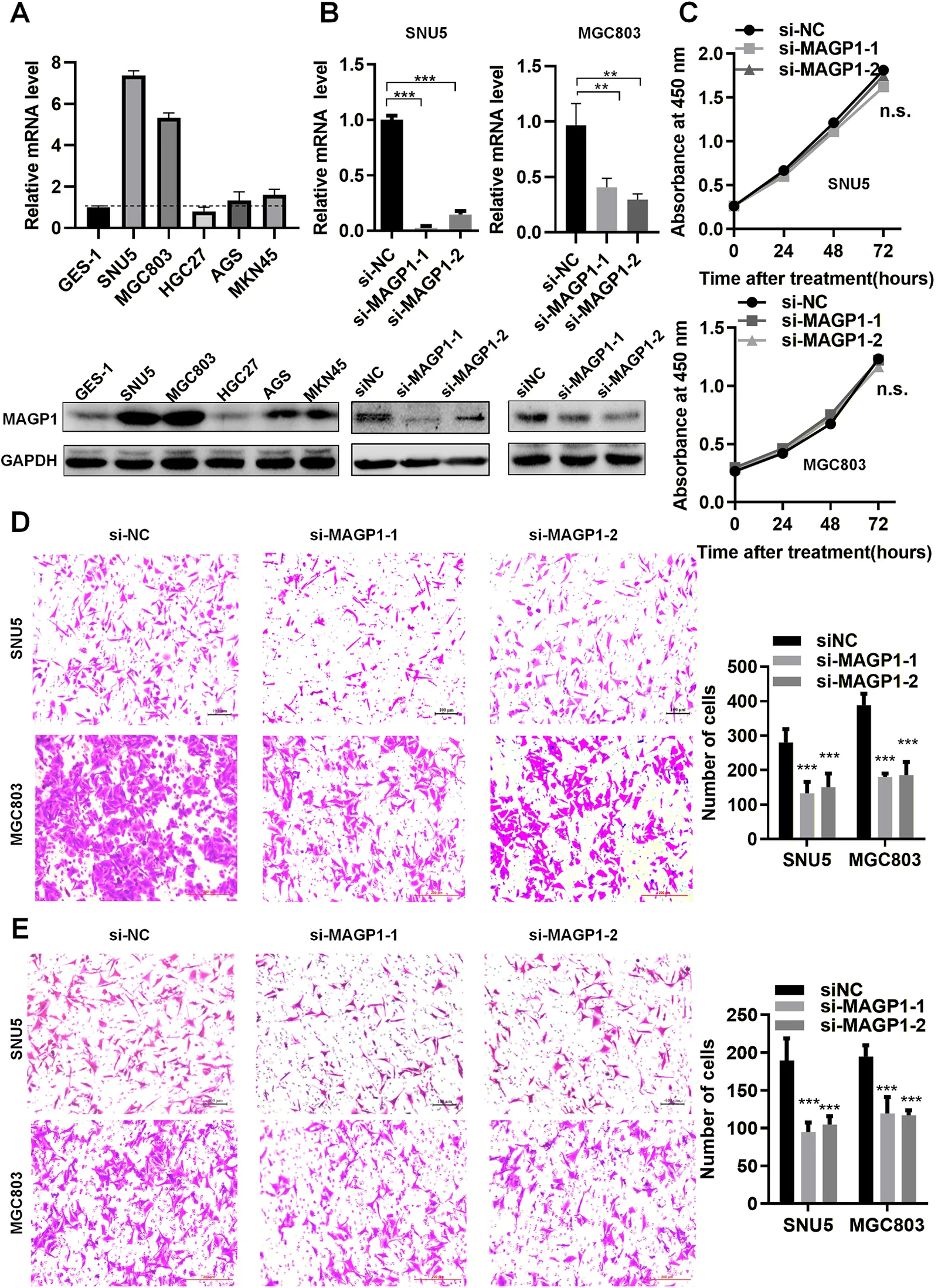
MAGP1 regulates migration and invasion of human GC cell lines. **(A)** mRNA and protein expression levels of MAGP1 were checked in a panel of five human GC cell lines and normal gastric cells (GES-1) using RT-qPCR and immunoblotting. **(B)** mRNA and protein expression level of MAGP1 were efficiently inhibited by si-MAGP1–1 and si-MAGP1–2 in SNU5 and MGC803 cells. **(C)** CCK-8 assay, **(D)** migration assay, and **(E)** invasion assay were performed in SNU5 and MGC803 cells transfected with si-MAGP1-1, si-MAGP1–2, and control siRNA (**P < 0.01; ***P < 0.001).

The original version of this article has been updated.

