# Supplementary material for: Overexpressed MAGP1 Is Associated With a Poor Prognosis and Promotes Cell Migration and Invasion in Gastric Cancer
Source: Front Oncol. 2020 Jan 17;9:1544. doi: 10.3389/fonc.2019.01544 (PMC6978879; doi:10.3389/fonc.2019.01544)
Supplement: Table S1 — The summary of 60 putative biomarkers. [file Table_1.docx]

**Table S1. The summary of 60 putative biomarkers**

| Gene name | Gene ID | Kaplan-Meier plotter | | TCGA | | Gene function Summary | Cancer related studies |
| --- | --- | --- | --- | --- | --- | --- | --- |
|  |  | ***P* Value** | ***P* Value** | ***P* Value** | ***P* Value** |  |  |
| ABCA8 (ATP binding cassette subfamily A member 8) | 10351 | 0.0313 | 0.2214 | 0.00088 | 0.00053 | ABCA8 (ATP Binding Cassette Subfamily A Member 8) is a Protein Coding gene. Among its related pathways are CDK-mediated phosphorylation and removal of Cdc6 and Transport of glucose and other sugars, bile salts and organic acids, metal ions and amine compounds. Gene Ontology (GO) annotations related to this gene include GTP binding and ATPase activity. An important paralog of this gene is ABCA9. | Dvorak P, et al. found that ABC gene expression profiles have clinical importance and possibly form a new hallmark of cancer[^1^](#_ENREF_1).  Demidenko R, et al. suggested that frequent down-regulation of the ABC transporter genes in prostate cancer [^2^](#_ENREF_2).  Xu CQ, et al. revealed ABCA8 might play critical roles in regulating the expression of human esophageal squamous cell carcinoma[^3^](#_ENREF_3).  Hedditch EL, et al. showed that expression of ABCA transporters was associated with poor outcome in serous ovarian cancer[^4^](#_ENREF_4)^,^[^5^](#_ENREF_5). |
| AEBP1 (AE binding protein 1) | 165 | 0.000000063 | 0.0004 | 0.12 | 0.11 | AEBP1 is a Protein Coding gene. AEBP1 may positively regulate MAP-kinase activity in adipocytes, leading to enhanced adipocyte proliferation and reduced adipocyte differentiation. And it also positively regulate NF-kappa-B activity in macrophages by promoting the phosphorylation and subsequent degradation of I-kappa-B-alpha (NFKBIA), leading to enhanced macrophage inflammatory responsiveness. AEBP1 Can act as a transcriptional repressor. | Ladha J, et al. identified the genomic targets of transcription factor AEBP1 and its role in survival of glioma cells[^6^](#_ENREF_6).  Hu W, et al. revealed AEBP1 may represent a novel therapeutic target for treating BRAF inhibitor-resistant melanoma[^7^](#_ENREF_7). |
| ANTXR1 (anthrax toxin receptor 1) | 84168 | 0.0212 | 0.0202 | 0.086 | 0.0058 | This gene encodes a type I transmembrane protein. Diseases associated with ANTXR1 include Gapo Syndrome and Tetramelic Deficiencies, Ectodermal Dysplasia, Deformed Ears, And Other Abnormalities. Among its related pathways are Cellular roles of Anthrax toxin and HIV Life Cycle. Gene Ontology (GO) annotations related to this gene include receptor activity and actin filament binding. An important paralog of this gene is ANTXR2. | Cao C, et al. found that down-regulation of tumor endothelial marker 8 suppresses cell proliferation mediated by ERK1/2 activity[^8^](#_ENREF_8).  Byrd TT, et al. suggested TEM8/ANTXR1-Specific CAR T Cells might be as a Targeted Therapy for Triple-Negative Breast Cancer[^9^](#_ENREF_9).  Gong Q, et al. found effect of silencing TEM8 gene on proliferation, apoptosis, migration and invasion of XWLC‑05 lung cancer cells[^10^](#_ENREF_10).  Chen D, et al. found ANTXR1, a stem cell-enriched functional biomarker, connects collagen signaling to cancer stem-like cells and metastasis in breast cancer[^11^](#_ENREF_11). |
| AOX1 (aldehyde oxidase 1) | 316 | 0.089 | 0.5236 | 0.091 | 0.044 | AOX1 is a Protein Coding gene. Diseases associated with AOX1 include Xanthinuria and Xanthinuria, Type Ii. Among its related pathways are Phenylalanine metabolism and superpathway of tryptophan utilization. Gene Ontology (GO) annotations related to this gene include oxidoreductase activity and electron transfer activity. An important paralog of this gene is XDH. | Haldrup C, et al. found AOX1may be a DNA methylation signature for prediction of biochemical recurrence after radical prostatectomy of clinically localized prostate cancer[^12^](#_ENREF_12)^,^ [^13^](#_ENREF_13). |
| ATP8B2 (ATPase phospholipid transporting 8B2) | 57198 | 0.0003 | 0.0007 | 0.029 | 0.13 | ATP8B2 (ATPase Phospholipid Transporting 8B2) is a Protein Coding gene. Among its related pathways are Mesodermal Commitment Pathway and Ion channel transport. Gene Ontology (GO) annotations related to this gene include nucleotide binding and cation-transporting ATPase activity. An important paralog of this gene is ATP8B4. | Few studies were reported. |
| BICC1 (BicC family RNA binding protein 1) | 80114 | 3.7E-13 | 1.9E-09 | 0.0041 | 0.26 | This gene encodes an RNA-binding protein that is active in regulating gene expression by modulating protein translation during embryonic development. | Few studies were reported. |
| C1R (complement C1r) | 715 | 0.0002 | 0.0058 | 0.18 | 0.95 | This gene is a Protein Coding gene. Diseases associated with C1R include Ehlers-Danlos Syndrome, Periodontal Type, 1 and Periodontal Ehlers-Danlos Syndrome.Gene Ontology (GO) annotations related to this gene include calcium ion binding and serine-type peptidase activity. An important paralog of this gene is MASP2. | Božić T, et al. found that DNA-methylation in C1R is a prognostic biomarker for acute myeloid leukemia[^14^](#_ENREF_14). |
| C1S (complement C1s) | 716 | 0.0002 | 0.022 | 0.34 | 0.52 | This gene encodes a serine protease, which is a major constituent of the human complement subcomponent C1. C1s associates with two other complement components C1r and C1q in order to yield the first component of the serum complement system. | Few studies were reported. |
| CERCAM (cerebral endothelial cell adhesion molecule) | 51148 | 0.00000026 | 0.0005 | 0.42 | 0.43 | This gene is a Protein Coding gene,Probable cell adhesion protein involved in leukocyte transmigration across the blood-brain barrier. | Few studies were reported. |
| COL10A1 (collagen type X alpha 1 chain) | 1300 | 0.0443 | 0.5073 | 0.084 | 0.18 | This gene encodes the alpha chain of type X collagen, a short chain collagen expressed by hypertrophic chondrocytes during endochondral ossification. Unlike type VIII collagen, the other short chain collagen, type X collagen is a homotrimer. | Chapman KB, et al. revealed COL10A1 expression is elevated in diverse solid tumor types and is associated with tumor vasculature[^15^](#_ENREF_15).  Huang H, et al. found high expression of COL10A1 is associated with poor prognosis in colorectal cancer[^16^](#_ENREF_16). |
| CPA3 (carboxypeptidase A3) | 1359 | 0.7384 | 0.3779 | 0.16 | 0.017 | This gene encodes a member of the carboxypeptidase A family of zinc metalloproteases. The encoded preproprotein is proteolytically processed to generate a mature protease that is released by mast cells and may be involved in the degradation of endogenous proteins and the inactivation of venom-associated peptides. | Huang H,et al. showed that Carboxypeptidase A3 (CPA3): a novel gene highly induced by histone deacetylase inhibitors during differentiation of prostate epithelial cancer cells[^17^](#_ENREF_17). |
| CPVL (carboxypeptidase, vitellogenic like) | 54504 | 0.3948 | 0.7481 | 0.11 | 0.23 | The protein encoded by this gene is a carboxypeptidase and bears strong sequence similarity to serine carboxypeptidases. Carboxypeptidases are a large class of proteases that act to cleave a single amino acid from the carboxy termini of proteins or peptides. The exact function of this protein, however, has not been determined. | Few studies were reported. |
| CPZ (carboxypeptidase Z) | 8532 | 0.0096 | 0.25 | 0.014 | 0.017 | This gene encodes a member of the metallocarboxypeptidase family. This enzyme displays carboxypeptidase activity towards substrates with basic C-terminal residues. It is most active at neutral pH and is inhibited by active site-directed inhibitors of metallocarboxypeptidases. Alternative splicing in the coding region results in multiple transcript variants encoding different isoforms. | Few studies were reported. |
| CTSK (cathepsin K) | 1513 | 0.3931 | 0.6588 | 0.43 | 0.73 | The protein encoded by this gene is a lysosomal cysteine proteinase involved in bone remodeling and resorption. This protein, which is a member of the peptidase C1 protein family, is predominantly expressed in osteoclasts. However, the encoded protein is also expressed in a significant fraction of human breast cancers, where it could contribute to tumor invasiveness. | Brubaker KD ,et al. showed that Cathepsin K mRNA and protein expression in prostate cancer progression[^18^](#_ENREF_18).  Leusink FK，et al. found upregulation of CTSK seems to be associated with high incidence of lymphatic spread and poor survival in oral squamous cell carcinoma[^19^](#_ENREF_19).  Fan X, et al. revealed elevated Cathepsin K potentiates metastasis of epithelial ovarian cancer[^20^](#_ENREF_20). |
| DCN (decorin) | 1634 | 0.3059 | 0.622 | 0.015 | 0.0043 | This gene encodes a member of the small leucine-rich proteoglycan family of proteins. Alternative splicing results in multiple transcript variants, at least one of which encodes a preproprotein that is proteolytically processed to generate the mature protein. This protein plays a role in collagen fibril assembly. | Nyman MC,et al. found that Decorin in Human Colon Cancer: Localization In Vivo and Effect on Cancer Cell Behavior In Vitro[^21^](#_ENREF_21).  Zhang W, et al. found DCN plays vital roles in cancer cell proliferation, spread, pro-inflammatory processes and anti-fibrillogenesis^[22](#_ENREF_22)^. |
| DEGS2 (delta 4-desaturase, sphingolipid 2) | 123099 | 0.901 | 0.1601 | 0.028 | 0.055 | This gene encodes a bifunctional enzyme that is involved in the biosynthesis of phytosphingolipids in human skin and in other phytosphingolipid-containing tissues. This enzyme can act as a sphingolipid delta(4)-desaturase, and also as a sphingolipid C4-hydroxylase. | Few studies were reported. |
| EFEMP1 (EGF containing fibulin extracellular matrix protein 1) | 2202 | 0.0412 | 0.3825 | 0.12 | 0.14 | This gene encodes a member of the fibulin family of extracellular matrix glycoproteins. Like all members of this family, the encoded protein contains tandemly repeated epidermal growth factor-like repeats followed by a C-terminus fibulin-type domain. This gene is upregulated in malignant gliomas and may play a role in the aggressive nature of these tumors. | Shen H,et al. revealed epidermal Growth Factor-Containing Fibulin-Like Extracellular Matrix Protein 1 (EFEMP1) Acts as a Potential Diagnostic Biomarker for Prostate Cancer[^23^](#_ENREF_23).  Yin X, et al. found EFEMP1 promotes ovarian cancer cell growth, invasion and metastasis via activated the AKT pathway[^24^](#_ENREF_24). |
| FAP (fibroblast activation protein alpha) | 2191 | 0.1672 | 0.327 | 0.23 | 0.48 | The protein encoded by this gene is a homodimeric integral membrane gelatinase belonging to the serine protease family. It is selectively expressed in reactive stromal fibroblasts of epithelial cancers, granulation tissue of healing wounds, and malignant cells of bone and soft tissue sarcomas. | Liao Y, et al. showed evaluation of the circulating level of fibroblast activation protein α for diagnosis of esophageal squamous cell carcinoma[^25^](#_ENREF_25).  da Silva AC, et al. found FAP stainings 2/3 was significantly related to histological grades 2 and 3 in ovarian cancer[^26^](#_ENREF_26).  Yang L, et al. found over-expression of fibroblast activation protein alpha increases tumor growth in xenografts of ovarian cancer cells[^27^](#_ENREF_27). |
| FBLN2 (fibulin 2) | 2199 | 0.0182 | 0.1829 | 0.26 | 0.079 | FBLN2 (Fibulin 2) is a Protein Coding gene.Diseases associated with FBLN2 include Familial Osteochondritis Dissecans and Osteochondritis Dissecans. Among its related pathways are Cell adhesion_Cell-matrix glycoconjugates and Elastic fibre formation. Gene Ontology (GO) annotations related to this gene include calcium ion binding and extracellular matrix binding. An important paralog of this gene is FBLN1. | Law EW,et al. found anti-angiogenic and tumor-suppressive roles of candidate tumor-suppressor gene, Fibulin-2, in nasopharyngeal carcinoma[^28^](#_ENREF_28).  Baird BN, et al. found Fibulin-2 is a driver of malignant progression in lung adenocarcinoma[^29^](#_ENREF_29).  Yi CH, et al. found loss of fibulin-2 expression is associated with breast cancer progression[^30^](#_ENREF_30). |
| FBLN5 (fibulin 5) | 10516 | 0.0000036 | 0.0001 | 0.012 | 0.0057 | The protein encoded by this gene is a secreted, extracellular matrix protein containing an Arg-Gly-Asp (RGD) motif and calcium-binding EGF-like domains. It promotes adhesion of endothelial cells through interaction of integrins and the RGD motif. It is prominently expressed in developing arteries but less so in adult vessels. | Heo JH, et al. showed Fibulin-5 is a tumour suppressor inhibiting cell migration and invasion in ovarian cancer[^31^](#_ENREF_31).  Manders DB, et al. found dysregulation of fibulin-5 and matrix metalloproteases in epithelial ovarian cancer[^32^](#_ENREF_32).  Shi XY, et al. found Fibulin-5 may act as a key factor in the progression of gastric cancer[^33^](#_ENREF_33). |
| FBN1 (fibrillin 1) | 2200 | 0.2685 | 0.4903 | 0.07 | 0.32 | This gene encodes a member of the fibrillin family of proteins. The encoded preproprotein is proteolytically processed to generate two proteins including the extracellular matrix component fibrillin-1 and the protein hormone asprosin. Fibrillin-1 is an extracellular matrix glycoprotein that serves as a structural component of calcium-binding microfibrils. | Guo Q,et al. found detection of hypermethylated fibrillin-1 in the stool samples of colorectal cancer patients[^34^](#_ENREF_34).  Zhu J, et al. found Fibulin-1 protein expression may be useful as a diagnosis and prognosis marker for colorectal cancer[^35^](#_ENREF_35).  Cui Y, et al. found fibulin-1 was an independent prognostic marker for lung adenocarcinoma[^36^](#_ENREF_36). |
| FGF7 (fibroblast growth factor 7) | 2252 | 0.1165 | 0.8502 | 0.013 | 0.15 | The protein encoded by this gene is a member of the fibroblast growth factor (FGF) family. FGF family members possess broad mitogenic and cell survival activities, and are involved in a variety of biological processes, including embryonic development, cell growth, morphogenesis, tissue repair, tumor growth and invasion. | Fan EW, et al. found that FGF7 over expression is an Independent Prognosticator in Patients with Urothelial Carcinoma of the Upper Urinary Tract and Bladder[^37^](#_ENREF_37).  Huang T, et al. found FGF7 promotes invasion and migration in human gastric cancer[^38^](#_ENREF_38). |
| GAS1 (growth arrest specific 1) | 2619 | 0.0008 | 0.0109 | 0.11 | 0.93 | Growth arrest-specific 1 plays a role in growth suppression. GAS1 blocks entry to S phase and prevents cycling of normal and transformed cells. Gas1 is a putative tumor suppressor gene. | Conceição AL, et al. showed that downregulation of OCLN and GAS1 in clear cell renal cell carcinoma[^39^](#_ENREF_39).  Li Q, et al. found Gas1 Inhibits Metastatic and Metabolic Phenotypes in Colorectal Carcinoma[^40^](#_ENREF_40). |
| GFPT2 (glutamine-fructose-6-phosphate transaminase 2) | 9945 | 0.0000091 | 0.0003 | 0.075 | 0.13 | This gene is a Protein Coding gene,Diseases associated with GFPT2 include Pancreatic Steatorrhea and Lipodystrophy,Gene Ontology (GO) annotations related to this gene include carbohydrate binding and glutamine-fructose-6-phosphate transaminase (isomerizing) activity. An important paralog of this gene is GFPT1. | Few studies were reported. |
| GGT5 (gamma-glutamyltransferase 5) | 2687 | 6E-11 | 5.9E-09 | 0.008 | 0.045 | The protein encoded by this gene is synthesized as a single, catalytically-inactive polypeptide, that is processed post-transcriptionally to form a heavy and light subunit, with the catalytic activity contained within the small subunit. | Few studies were reported. |
| GNB4 (G protein subunit beta 4) | 59345 | 0.0083 | 0.0031 | 0.047 | 0.27 | Heterotrimeric guanine nucleotide-binding proteins (G proteins), which integrate signals between receptors and effector proteins, are composed of an alpha, a beta, and a gamma subunit.This gene encodes a beta subunit. Beta subunits are important regulators of alpha subunits, as well as of certain signal transduction receptors and effectors. | Riemann K,et al. found the association of GNB4 intron-1 haplotypes with survival in patients with UICC stage III and IV colorectal carcinoma[^41^](#_ENREF_41). |
| GPNMB (glycoprotein nmb) | 10457 | 0.1757 | 0.1403 | 0.0011 | 0.37 | The protein is a melanocyte-specific protein. GPNMB shows expression in the lowly metastatic human melanoma cell lines and xenografts but does not show expression in the highly metastatic cell lines. GPNMB may be involved in growth delay and reduction of metastatic potential. | Smuczek B, et al.revealedthe laminin-derived peptide C16 regulates GPNMB expression and function in breast cancer[^42^](#_ENREF_42).  Jin R, et al. found GPNMB promotes the proliferation and metastasis of osteosarcoma cells[^43^](#_ENREF_43). |
| GPX8 (glutathione peroxidase 8) | 493869 | 0.000079 | 0.0002 | 0.11 | 0.055 | This gene is a Protein Coding gene. Among its related pathways are Detoxification of Reactive Oxygen Species and Aldosterone synthesis and secretion. Gene Ontology (GO) annotations related to this gene include oxidoreductase activity and peroxidase activity. | Few studies were reported. |
| GREM1 (gremlin 1, DAN family BMP antagonist) | 26585 | 0.0156 | 0.7178 | 0.063 | 0.87 | This gene may play a role in regulating organogenesis, body patterning, and tissue differentiation. In mouse, this protein has been shown to relay the sonic hedgehog (SHH) signal from the polarizing region to the apical ectodermal ridge during limb bud outgrowth. Alternatively spliced transcript variants encoding different isoforms have been found for this gene. | Honma R,et al.foundclinicopathological and Prognostic Significance of Epithelial Gremlin1 Expression in Gastric Cancer[^44^](#_ENREF_44). |
| ID1 (inhibitor of DNA binding 1, HLH protein) | 3397 | 0.1521 | 0.3889 | 0.11 | 0.44 | The encoded protein has no DNA binding activity and therefore can inhibit the DNA binding and transcriptional activation ability of basic HLH proteins with which it interacts. This protein may play a role in cell growth, senescence, and differentiation. | Li DN, et al. showed expression of ID1 and its effects on angiogenesis in gastric cancer[^45^](#_ENREF_45).  X Yin, et al. found ID1 promotes hepatocellular carcinoma proliferation and confers chemoresistance to oxaliplatin[^46^](#_ENREF_46). |
| ISLR (immunoglobulin superfamily containing leucine rich repeat) | 3671 | 0.1744 | 0.6083 | 0.05 | 0.073 | A Protein Coding gene. Among its related pathways are Response to elevated platelet cytosolic Ca2+. | Few studies were reported. |
| ITGBL1 (integrin subunit beta like 1) | 9358 | 4.90E-09 | 9.00E-07 | 0.073 | 0.81 | This gene encodes a beta integrin-related protein that is a member of the EGF-like protein family. The encoded protein contains integrin-like cysteine-rich repeats. | Gan, X et al.found epigenetic downregulated ITGBL1 promotes non-small cell lung cancer cell invasion through Wnt/PCP signaling[^47^](#_ENREF_47).  Li, R et al. found ITGBL1 Predicts a Poor Prognosis and Correlates EMT Phenotype in Gastric Cancer[^48^](#_ENREF_48).  Sun, L et al. found extracellular matrix protein ITGBL1 promotes ovarian cancer cell migration and adhesion through Wnt/PCP signaling and FAK/SRC pathway[^49^](#_ENREF_49). |
| LAMA2 (laminin subunit alpha 2) | 3908 | 4.40E-05 | 0.0055 | 0.023 | 0.052 | Protein encoded by this gene is a major component of the basement membrane. It is thought to mediate the attachment, migration, and organization of cells into tissues during embryonic development by interacting with other extracellular matrix components. | Lee, S et al. identified GABRA1 and LAMA2 as new DNA methylation markers in colorectal cancer[^50^](#_ENREF_50). |
| LUM (lumican) | 4060 | 0.1137 | 0.4366 | 0.041 | 0.27 | A Protein Coding gene.GO annotations related to this gene include collagen binding and extracellular matrix structural constituent. | de Wit, M et al. found lumican and versican protein expression are associated with colorectal adenoma-to-carcinoma progression[^51^](#_ENREF_51).  Li, X et al. found prolonged exposure to extracellular lumican restrains pancreatic adenocarcinoma growth[^52^](#_ENREF_52).  Wang, X et al found Cancer-associated fibroblast-derived Lumican promotes gastric cancer progression[^53^](#_ENREF_53). |
| MEOX2 (mesenchyme homeobox 2) | 4223 | 0.0003 | 0.0618 | 0.011 | 0.091 | A Protein Coding gene.GO annotations related to this gene include DNA binding transcription factor activity and RNA polymerase II proximal promoter sequence-specific DNA binding. | Chen, Y et al. found MEOX2 regulates nuclear factor-kappaB activity in vascular endothelial cells through interactions with p65 and IkappaBbeta^[54](#_ENREF_54)^. |
| MAGP1 (microfibril associated glycoprotein 1) | 4237 | 0.00004 | 0.0017 | 0.019 | 0.017 | A Protein Coding gene.GO annotations related to this gene include fibronectin binding and fibrinogen binding.MAGP1 is a protective factor for obesity and diabetes and promotes thermogenesis by regulating TGF-β/Smad3 signaling.Loss of MAGP1 can affect the development of caudal blood vessel in zebrafish | Craft C S, et al. found the Extracellular Matrix Protein MAGP1 supports thermogenesis and protects against obesity and diabetes through regulation of TGF-β[^55^](#_ENREF_55).  Weinbaum JS, et al. revealed deficiency in microfibril-associated glycoprotein-1 leads to complex phenotypes in multiple organ systems[^56^](#_ENREF_56).  Chen E, et al. showed functional analysis of zebrafish microfibril-associated glycoprotein-1 (Magp1) in vivo reveals roles for microfibrils in vascular development and function[^57^](#_ENREF_57). |
| MAGP2 (microfibril associated glycoprotein 2) | 8076 | 0.0005 | 0.0724 | 0.16 | 0.13 | A Protein Coding gene.Among its related pathways are MFAP5-mediated ovarian cancer cell motility and invasiveness and Elastic fibre formation. Gene Ontology (GO) annotations related to this gene include extracellular matrix structural constituent. | Leung, C. S et al. found calcium-dependent FAK/CREB/TNNC1 signalling mediates the effect of stromal MFAP5 on ovarian cancer metastatic potential[^58^](#_ENREF_58). |
| MYH10 (myosin heavy chain 10) | 4628 | 5.90E-07 | 7.90E-06 | 0.12 | 0.15 | A Protein Coding gene.Among its related pathways are PAK Pathway and EPH-Ephrin signaling. | Antony-Debre, I et al. found MYH10 protein expression in platelets as a biomarker of RUNX1 and FLI1 alterations[^59^](#_ENREF_59). |
| NCAM2 (neural cell adhesion molecule 2) | 4685 | 0.0392 | 0.5362 | 0.012 | 0.64 | The protein encoded by this gene belongs to the immunoglobulin superfamily. It is a type I membrane protein and may function in selective fasciculation and zone-to-zone projection of the primary olfactory axons. | Few studies were reported. |
| TENM3 (teneurin transmembrane protein 3) | 55714 | 0.0173 | 0.2245 | 0.12 | 0.19 | This gene encodes a large transmembrane protein that may be involved in the regulation of neuronal development. | Few studies were reported. |
| PMP22 (peripheral myelin protein 22) | 5376 | 0.0017 | 0.0022 | 0.79 | 0.94 | This gene encodes an integral membrane protein that is a major component of myelin in the peripheral nervous system. Studies suggest two alternately used promoters drive tissue-specific expression. | Tong, D et al. identified PMP22 as an independent prognostic factor for disease-free and overall survival in breast cancer patients[^60^](#_ENREF_60).  Qu, H et al. found Suppression of PMP22 expression by miR29 inhibits the progression of lung cancer[^61^](#_ENREF_61).  Cai, W et al. found PMP22 Regulates Self-Renewal and Chemoresistance of Gastric Cancer Cells[^62^](#_ENREF_62). |
| PRICKLE1 (prickle planar cell polarity protein 1) | 144165 | 4.6E-09 | 0.0000055 | 0.045 | 0.015 | his gene encodes a nuclear receptor that may be a negative regulator of the Wnt/beta-catenin signaling pathway. The encoded protein localizes to the nuclear membrane and has been implicated in the nuclear trafficking of the transcription repressors REST/NRSF and REST4. | Chan, D. W et al. found prickle-1 negatively regulates Wnt/beta-catenin pathway by promoting Dishevelled ubiquitination/degradation in liver cancer[^63^](#_ENREF_63). |
| PROM2 (prominin 2) | 150696 | 0.8057 | 0.383 | 0.067 | 0.75 | This gene encodes a member of the prominin family of pentaspan membrane glycoproteins. The encoded protein localizes to basal epithelial cells and may be involved in the organization of plasma membrane microdomains. | Singh, R. D et al. found Prominin-2 expression increases protrusions, decreases caveolae and inhibits Cdc42 dependent fluid phase endocytosis[^64^](#_ENREF_64). |
| PRRX1 (paired related homeobox 1) | 5396 | 0.0168 | 0.011 | 0.058 | 0.11 | The DNA-associated protein encoded by this gene is a member of the paired family of homeobox proteins localized to the nucleus. The protein functions as a transcription co-activator, enhancing the DNA-binding activity of serum response factor, a protein required for the induction of genes by growth and differentiation factors. The protein regulates muscle creatine kinase, indicating a role in the establishment of diverse mesodermal muscle types. | Takano, S et al found Prrx1 isoform switching regulates pancreatic cancer invasion and metastatic colonization[^65^](#_ENREF_65).  Hirata, H et al. found downregulation of PRRX1 Confers Cancer Stem Cell-Like Properties and Predicts Poor Prognosis in Hepatocellular Carcinoma[^66^](#_ENREF_66).  Takahashi, Y et al. found paired related homoeobox 1, a new EMT inducer, is involved in metastasis and poor prognosis in colorectal cancer[^67^](#_ENREF_67). |
| PTGER3 (prostaglandin E receptor 3) | 5733 | 0.0197 | 0.2519 | 0.3 | 0.042 | A Protein Coding gene.Among its related pathways are Peptide ligand-binding receptors and cAMP signaling pathway. Gene Ontology (GO) annotations related to this gene include G-protein coupled receptor activity and prostaglandin E receptor activity. | Kashiwagi, E et al found PTGER3 mediates growth inhibitory effect of aspirin through androgen receptor and contributes to castration resistance in prostate cancer cells[^68^](#_ENREF_68).  Heidegger, H et al. identified PTGER3 as an Independent Negative Prognostic Factor for Cervical Cancer Patients[^69^](#_ENREF_69). |
| PTPRN (protein tyrosine phosphatase, receptor type N) | 5798 | 0.013 | 0.1279 | 0.6 | 0.66 | The protein encoded by this gene is a member of the protein tyrosine phosphatase (PTP) family. PTPs are known to be signaling molecules that regulate a variety of cellular processes including cell growth, differentiation, mitotic cycle, and oncogenic transformation. | Few studies were reported. |
| RAB31 (RAB31, member RAS oncogene family) | 11031 | 0.0002 | 0.0005 | 0.028 | 0.18 | A Protein Coding gene. Diseases associated with RAB31 include Estrogen-Receptor Positive Breast Cancer. Among its related pathways are Metabolism of proteins and Innate Immune System. | Sui, Y et al. found Rab31 promoted hepatocellular carcinoma (HCC) progression via inhibition of cell apoptosis induced by PI3K/AKT/Bcl-2/BAX pathway[^70^](#_ENREF_70).  Jin, C et al. found cooperative interaction between the MUC1-C oncoprotein and the Rab31 GTPase in estrogen receptor-positive breast cancer cells[^71^](#_ENREF_71).  Kotzsch, M et al. found mRNA expression levels of the biological factor rab31, displaying prognostic value in breast cancer, are not clinically relevant in advanced ovarian cancer[^72^](#_ENREF_72). |
| SERPINE2 (serpin family E member 2) | 5270 | 3.70E-06 | 0.0145 | 0.072 | 0.21 | This gene encodes a member of the serpin family of proteins, a group of proteins that inhibit serine proteases. Thrombin, urokinase, plasmin and trypsin are among the proteases that this family member can inhibit. This gene is a susceptibility gene for chronic obstructive pulmonary disease and for emphysema. | Wang, K et al. found upregulated SERPINE2 may contribute to the aggressive phenotype of gastric cancer[^73^](#_ENREF_73).  An, L et al. found the interaction among EPHX1, GSTP1, SERPINE2, and TGFB1 contributing to the quantitative traits of chronic obstructive pulmonary disease in Chinese Han population[^74^](#_ENREF_74). |
| SERPINF1 (serpin family F member 1) | 5176 | 1.00E-04 | 0.0247 | 0.028 | 0.1 | A Protein Coding gene.Among its related pathways are Apoptotic Pathways in Synovial Fibroblasts and Wnt Signaling Pathway and Pluripotency. | Few studies were reported. |
| SERPING1 (serpin family G member 1) | 710 | 0.0002 | 0.0046 | 0.24 | 0.14 | This gene encodes a highly glycosylated plasma protein involved in the regulation of the complement cascade. Its protein inhibits activated C1r and C1s of the first complement component and thus regulates complement activation. Deficiency of this protein is associated with hereditary angioneurotic oedema (HANE). | Few studies were reported. |
| SFRP4 (secreted frizzled related protein 4) | 6424 | 0.0137 | 0.9532 | 0.4 | 0.47 | A Protein Coding gene.Among its related pathways are Wnt Signaling Pathway and Pluripotency and Wnt Signaling Pathways: beta-Catenin-dependent Wnt Signaling. Gene Ontology (GO) annotations related to this gene include G-protein coupled receptor activity and Wnt-protein binding. | Sandsmark, E et al. found SFRP4 gene expression is increased in aggressive prostate cancer[^75^](#_ENREF_75).  Deshmukh, A et al. found secreted Frizzled-related protein 4 (sFRP4) chemo-sensitizes cancer stem cells derived from human breast, prostate, and ovary tumor cell lines[^76^](#_ENREF_76). |
| SLC2A3 (solute carrier family 2 member 3) | 6515 | 0.2219 | 0.497 | 0.0017 | 0.23 | A Protein Coding gene.Gene Ontology (GO) annotations related to this gene include transmembrane transporter activity and glucose transmembrane transporter activity. | Few studies were reported. |
| SLIT2 (slit guidance ligand 2) | 9353 | 3.70E-05 | 0.0925 | 0.11 | 0.13 | This gene encodes a member of the slit family of secreted glycoproteins, which are ligands for the Robo family of immunoglobulin receptors. Slit proteins play highly conserved roles in axon guidance and neuronal migration and may also have functions during other cell migration processes including leukocyte migration. | Shi, R et al. found knockdown of Slit2 promotes growth and motility in gastric cancer cells via activation of AKT/beta-catenin[^77^](#_ENREF_77).  Qin, F et al. found Low Expression of Slit2 and Robo1 is Associated with Poor Prognosis and Brain-specific Metastasis of Breast Cancer Patients[^78^](#_ENREF_78). |
| SPON1 (spondin 1) | 10418 | 0.039 | 0.8444 | 0.027 | 0.018 | A Protein Coding gene.Among its related pathways are Apoptotic Pathways in Synovial Fibroblasts and PAK Pathway. | Chang, H et al. found SPON1 promotes metastatic progression through Fak and Src dependent pathway in human osteosarcoma[^79^](#_ENREF_79). |
| SULF1 (sulfatase 1) | 23213 | 0.1056 | 0.9104 | 0.072 | 0.28 | A Protein Coding gene.Gene Ontology (GO) annotations related to this gene include calcium ion binding and arylsulfatase activity. | Liu, C. T et al. found SULF1 inhibits proliferation and invasion of esophageal squamous cell carcinoma cells by decreasing heparin-binding growth factor signaling[^80^](#_ENREF_80).  Lai, J. P et al. found SULF1 inhibits tumor growth and potentiates the effects of histone deacetylase inhibitors in hepatocellular carcinoma[^81^](#_ENREF_81). |
| SVEP1 (sushi, von Willebrand factor type A, EGF and pentraxin domain containing 10 | 79987 | 0.5434 | 0.3444 | 0.00035 | 0.0017 | A Protein Coding gene.Gene Ontology (GO) annotations related to this gene include calcium ion binding and chromatin binding. | Few studies were reported. |
| THBS4 (thrombospondin 4） | 7060 | 6.00E-06 | 0.1099 | 0.3 | 0.21 | The protein encoded by this gene belongs to the thrombospondin protein family. Thrombospondin family members are adhesive glycoproteins that mediate cell-to-cell and cell-to-matrix interactions. This protein forms a pentamer and can bind to heparin and calcium. It is involved in local signaling in the developing and adult nervous system, and it contributes to spinal sensitization and neuropathic pain states. This gene is activated during the stromal response to invasive breast cancer. It may also play a role in inflammatory responses in Alzheimer's disease. | Few studies were reported. |
| TIMP3 (TIMP metallopeptidase inhibitor 3) | 7078 | 0.0005 | 0.177 | 0.034 | 0.044 | This gene belongs to the TIMP gene family. The proteins encoded by this gene family are inhibitors of the matrix metalloproteinases, a group of peptidases involved in degradation of the extracellular matrix (ECM). Expression of this gene is induced in response to mitogenic stimulation and this netrin domain-containing protein is localized to the ECM. | Adissu, H. A et al. found Timp3 loss accelerates tumour invasion and increases prostate inflammation in a mouse model of prostate cancer[^82^](#_ENREF_82).  Han, X. G et al. found TIMP3 Overexpression Improves the Sensitivity of Osteosarcoma to Cisplatin by Reducing IL-6 Production[^83^](#_ENREF_83).  Maleva Kostovska, I et al. found TIMP3 Promoter Methylation Represents an Epigenetic Marker of BRCA1ness Breast Cancer Tumours^[84](#_ENREF_84)^. |
| WIPF1 (WAS/WASL interacting protein family member 1) | 7456 | 8.80E-07 | 0.0037 | 0.07 | 0.31 | This gene encodes a protein that plays an important role in the organization of the actin cytoskeleton. The encoded protein binds to a region of Wiskott-Aldrich syndrome protein that is frequently mutated in Wiskott-Aldrich syndrome, an X-linked recessive disorder. Impairment of the interaction between these two proteins may contribute to the disease. | Zhang, T et al. found epigenetically upregulated WIPF1 plays a major role in BRAF V600E-promoted papillary thyroid cancer aggressiveness[^85^](#_ENREF_85). |
| ZC3H12A (zinc finger CCCH-type containing 12A) | 80149 | 3.70E-07 | 6.90E-06 | 0.13 | 0.49 | ZC3H12A is an MCP1 (CCL2; MIM 158105)-induced protein that acts as a transcriptional activator and causes cell death of cardiomyocytes, possibly via induction of genes associated with apoptosis. | Few studies were reported. |

References:

[1] Dvorak P, Pesta M, Soucek P. ABC gene expression profiles have clinical importance and possibly form a new hallmark of cancer. Tumour Biology the Journal of the International Society for Oncodevelopmental Biology & Medicine. 2017; 39: 1010428317699800.

[2] Demidenko R, Razanauskas D, Daniunaite K, Lazutka JR, Jankevicius F, Jarmalaite S. Frequent down-regulation of ABC transporter genes in prostate cancer. Bmc Cancer. 2015; 15: 683.

[3] Xu CQ, Zhu ST, Wang M, et al. Pathway analysis of differentially expressed genes in human esophageal squamous cell carcinoma. European Review for Medical & Pharmacological Sciences. 2015; 19: 1652-61.

[4] Hedditch EL, Gao B, Russell AJ, et al. ABCA Transporter Gene Expression and Poor Outcome in Epithelial Ovarian Cancer. Journal of the National Cancer Institute. 2014; 106: 766-76.

[5] Liu X, Gao Y, Zhao B, et al. Discovery of microarray-identified genes associated with ovarian cancer progression. International Journal of Oncology. 2015; 46: 2467-78.

[6] Ladha J, Sinha S, Bhat V, Donakonda S, Rao SM. Identification of genomic targets of transcription factor AEBP1 and its role in survival of glioma cells. Molecular Cancer Research Mcr. 2012; 10: 1039.

[7] Hu W, Jin L, Jiang CC, et al. AEBP1 upregulation confers acquired resistance to BRAF (V600E) inhibition in melanoma. Cell Death & Disease. 2013; 4: e914.

[8] Cao C, Wang Z, Huang L, et al. Down-regulation of tumor endothelial marker 8 suppresses cell proliferation mediated by ERK1/2 activity. Sci Rep. 2016; 6: 23419.

[9] Byrd TT, Fousek K, Pignata A, et al. TEM8/ANTXR1-specific CAR T cells as a targeted therapy for triple-negative breast cancer. Cancer Research. 2017; 78: canres.1911.2016.

[10] Gong Q, Liu C, Wang C, Zhuang L, Zhang L, Wang X. Effect of silencing TEM8 gene on proliferation, apoptosis, migration and invasion of XWLC-05 lung cancer cells. Molecular Medicine Reports. 2018; 17: 911-7.

[11] Chen D, Bhat-Nakshatri P, Goswami C, Badve S, Nakshatri H. ANTXR1, a stem cell-enriched functional biomarker, connects collagen signaling to cancer stem-like cells and metastasis in breast cancer. Cancer Research. 2013; 73: 5821-33.

[12] Haldrup C, Mundbjerg K, Vestergaard EM, et al. DNA methylation signatures for prediction of biochemical recurrence after radical prostatectomy of clinically localized prostate cancer. Journal of Clinical Oncology Official Journal of the American Society of Clinical Oncology. 2013; 31: 3250-8.

[13] Møller M, Strand SH, Mundbjerg K, et al. Heterogeneous patterns of DNA methylation-based field effects in histologically normal prostate tissue from cancer patients. Sci Rep. 2017; 7: 40636.

[14] Božić T, Lin Q, Frobel J, et al. DNA-methylation in C1R is a prognostic biomarker for acute myeloid leukemia. Clinical Epigenetics. 2015; 7: 1-6.

[15] Chapman KB, Prendes MJ, Sternberg H, et al. COL10A1 expression is elevated in diverse solid tumor types and is associated with tumor vasculature. Future Oncology. 2012; 8: 1031-40.

[16] Huang H, Li T, Ye G, et al. High expression of COL10A1 is associated with poor prognosis in colorectal cancer. Oncotargets & Therapy. 2018; 11: 1571-81.

[17] Huang H, Reed CP, Zhang JS, Shridhar V, Wang L, Smith DI. Carboxypeptidase A3 (CPA3): a novel gene highly induced by histone deacetylase inhibitors during differentiation of prostate epithelial cancer cells. Cancer Research. 1999; 59: 2981-8.

[18] Brubaker KD, Vessella RL, True LD, Thomas R, Corey E. Cathepsin K mRNA and protein expression in prostate cancer progression. Journal of Bone & Mineral Research. 2003; 18: 222-30.

[19] Leusink FK, Koudounarakis E, Frank MH, Koole R, van Diest PJ, Willems SM. Cathepsin K associates with lymph node metastasis and poor prognosis in oral squamous cell carcinoma. Bmc Cancer. 2018; 18: 385.

[20] Fan X, Wang C, Song X, Liu H, Li X, Zhang Y. Elevated Cathepsin K potentiates metastasis of epithelial ovarian cancer. Histology & Histopathology. 2018: 11960.

[21] Nyman MC, Sainio AO, Pennanen MM, et al. Decorin in Human Colon Cancer: Localization In Vivo and Effect on Cancer Cell Behavior In Vitro. Journal of Histochemistry & Cytochemistry. 2015; 63: 710-20.

[22] Zhang W, Ge Y, Cheng Q, Zhang Q, Fang L, Zheng J. Decorin is a pivotal effector in the extracellular matrix and tumour microenvironment. Oncotarget. 2018; 9: 5480-91.

[23] Shen H, Zhang L, Zhou J, et al. Epidermal Growth Factor-Containing Fibulin-Like Extracellular Matrix Protein 1 (EFEMP1) Acts as a Potential Diagnostic Biomarker for Prostate Cancer. Med Sci Monit. 2017; 23: 216-22.

[24] Yin X, Fang S, Wang M, Wang Q, Fang R, Chen J. EFEMP1 promotes ovarian cancer cell growth, invasion and metastasis via activated the AKT pathway. Oncotarget. 2016; 7: 47938-53.

[25] Liao Y, Xing S, Xu B, Liu W, Zhang G. Evaluation of the circulating level of fibroblast activation protein Î± for diagnosis of esophageal squamous cell carcinoma. Oncotarget. 2017; 8: 30050.

[26] Da AS, Jammal MP, Etchebehere RM, Murta E, Nomelini RS. Role of Alpha-Smooth Muscle Actin and Fibroblast Activation Protein Alpha in Ovarian Neoplasms. Gynecologic & Obstetric Investigation. 2018.

[27] Lijuan, Yang, Dongmei. Over-expression of fibroblast activation protein alpha increases tumor growth in xenografts of ovarian cancer cells. 2013; 45: 928.

[28] Law EW, Cheung AK, Kashuba VI, et al. Anti-angiogenic and tumor-suppressive roles of candidate tumor-suppressor gene, Fibulin-2, in nasopharyngeal carcinoma. Oncogene. 2012; 31: 728-38.

[29] Baird BN, Schliekelman MJ, Ahn YH, et al. Fibulin-2 Is a Driver of Malignant Progression in Lung Adenocarcinoma. Plos One. 2013; 8: e67054.

[30] Yi CH, Smith DJ, West WW, Hollingsworth MA. Loss of fibulin-2 expression is associated with breast cancer progression. American Journal of Pathology. 2007; 170: 1535-45.

[31] Heo JH, Song JY, Jeong JY, et al. Fibulin-5 is a tumour suppressor inhibiting cell migration and invasion in ovarian cancer. Journal of Clinical Pathology. 2016; 69: 109.

[32] Manders DB, Kishore HA, Gazdar AF, et al. Dysregulation of fibulin-5 and matrix metalloproteases in epithelial ovarian cancer. Oncotarget. 2018; 9: 14251-67.

[33] Shi XY, Wang L, Cao CH, Li ZY, Chen J, Li C. Effect of Fibulin-5 on cell proliferation and invasion in human gastric cancer patients. Asian Pacific Journal of Tropical Medicine. 2014; 7: 787-91.

[34] Guo Q, Song Y, Zhang H, Wu X, Xia P, Dang C. Detection of hypermethylated fibrillin-1 in the stool samples of colorectal cancer patients. Medical Oncology. 2013; 30: 1-5.

[35] Zhu J, Chen R, Mo L, et al. Expression of fibulin-1 predicted good prognosis in patients with colorectal cancer. Am J Transl Res. 2015; 7: 339-47.

[36] Cui Y, Liu J, Yin HB, Liu YF, Liu JH. Fibulin-1 functions as a prognostic factor in lung adenocarcinoma. Japanese Journal of Clinical Oncology. 2015; 45: 854.

[37] Fan EW, Li CC, Wu WJ, et al. FGF7 Over Expression is an Independent Prognosticator in Patients with Urothelial Carcinoma of the Upper Urinary Tract and Bladder. Journal of Urology. 2015; 194: 223.

[38] Huang T, Lei W, Liu D, et al. FGF7/FGFR2 signal promotes invasion and migration in human gastric cancer through upregulation of thrombospondin-1. International Journal of Oncology. 2017; 50: 1501.

[39] Conceição AL, Da SC, Badial RM, et al. Downregulation of OCLN and GAS1 in clear cell renal cell carcinoma. Oncology Reports. 2017; 37: 1487.

[40] Li Q, Qin Y, Wei P, et al. Gas1 Inhibits Metastatic and Metabolic Phenotypes in Colorectal Carcinoma. Molecular Cancer Research. 2016; 14.

[41] Riemann K, Struwe H, Alakus H, et al. Association of GNB4 intron-1 haplotypes with survival in patients with UICC stage III and IV colorectal carcinoma. Anticancer Research. 2009; 29: 1271-4.

[42] Smuczek B, Santos ES, Siqueira AS, Jjv P, Freitas VM, Jaeger RG. The laminin-derived peptide C16 regulates GPNMB expression and function in breast cancer. Experimental Cell Research. 2017; 358: 323.

[43] Jin R, Jin YY, Tang YL, Yang HJ, Zhou XQ, Lei Z. GPNMB silencing suppresses the proliferation and metastasis of osteosarcoma cells by blocking the PI3K/Akt/mTOR signaling pathway. Oncology Reports. 2018.

[44] Honma R, Sakamoto N, Ishikawa A, et al. Clinicopathological and Prognostic Significance of Epithelial Gremlin1 Expression in Gastric Cancer. Anticancer Research. 2018; 38: 1419.

[45] Li DN, Wang L, Wang L, Li S, Wang YB. Expression of Inhibitor of Differentiation-1 and Its Effects on Angiogenesis in Gastric Cancer. Cancer Biother Radiopharm. 2016; 31: 233-7.

[46] Yin X, Tang B, Li JH, et al. ID1 promotes hepatocellular carcinoma proliferation and confers chemoresistance to oxaliplatin by activating pentose phosphate pathway. Journal of Experimental & Clinical Cancer Research. 2017; 36: 166.

[47] Gan X, Liu Z, Tong B, Zhou J. Epigenetic downregulated ITGBL1 promotes non-small cell lung cancer cell invasion through Wnt/PCP signaling. Tumour Biol. 2016; 37: 1663-9.

[48] Li R, Zhuang C, Jiang S, et al. ITGBL1 Predicts a Poor Prognosis and Correlates EMT Phenotype in Gastric Cancer. J Cancer. 2017; 8: 3764-73.

[49] Sun L, Wang D, Li X, Zhang L, Zhang H, Zhang Y. Extracellular matrix protein ITGBL1 promotes ovarian cancer cell migration and adhesion through Wnt/PCP signaling and FAK/SRC pathway. Biomed Pharmacother. 2016; 81: 145-51.

[50] Lee S, Oh T, Chung H, et al. Identification of GABRA1 and LAMA2 as new DNA methylation markers in colorectal cancer. Int J Oncol. 2012; 40: 889-98.

[51] de Wit M, Carvalho B, Delis-van Diemen PM, et al. Lumican and versican protein expression are associated with colorectal adenoma-to-carcinoma progression. PLoS One. 2017; 12: e0174768.

[52] Li X, Kang Y, Roife D, et al. Prolonged exposure to extracellular lumican restrains pancreatic adenocarcinoma growth. Oncogene. 2017; 36: 5432-8.

[53] Wang X, Zhou Q, Yu Z, et al. Cancer-associated fibroblast-derived Lumican promotes gastric cancer progression via the integrin beta1-FAK signaling pathway. Int J Cancer. 2017; 141: 998-1010.

[54] Chen Y, Rabson AB, Gorski DH. MEOX2 regulates nuclear factor-kappaB activity in vascular endothelial cells through interactions with p65 and IkappaBbeta. Cardiovasc Res. 2010; 87: 723-31.

[55] <MAGP1 the extracellular matrix and metabolism (1).pdf>.

[56] Weinbaum JS, Broekelmann TJ, Pierce RA, et al. Deficiency in microfibril-associated glycoprotein-1 leads to complex phenotypes in multiple organ systems. The Journal of biological chemistry. 2008; 283: 25533-43.

[57] Eleanor Chen JDL, and Stephen C. Ekker. Functional analysis of zebrafish microfibril-associated glycoprotein-1 (Magp1) in vivo reveals roles for microfibrils in vascular development and function. Blood. 2006; 107:

4364-4374.

[58] Leung CS, Yeung TL, Yip KP, et al. Calcium-dependent FAK/CREB/TNNC1 signalling mediates the effect of stromal MFAP5 on ovarian cancer metastatic potential. Nat Commun. 2014; 5: 5092.

[59] Antony-Debre I, Bluteau D, Itzykson R, et al. MYH10 protein expression in platelets as a biomarker of RUNX1 and FLI1 alterations. Blood. 2012; 120: 2719-22.

[60] Tong D, Heinze G, Pils D, et al. Gene expression of PMP22 is an independent prognostic factor for disease-free and overall survival in breast cancer patients. BMC Cancer. 2010; 10: 682.

[61] Qu H, Zhu M, Tao Y, Zhao Y. Suppression of peripheral myelin protein 22 (PMP22) expression by miR29 inhibits the progression of lung cancer. Neoplasma. 2015; 62: 881-6.

[62] Cai W, Chen G, Luo Q, et al. PMP22 Regulates Self-Renewal and Chemoresistance of Gastric Cancer Cells. Mol Cancer Ther. 2017; 16: 1187-98.

[63] Chan DW, Chan CY, Yam JW, Ching YP, Ng IO. Prickle-1 negatively regulates Wnt/beta-catenin pathway by promoting Dishevelled ubiquitination/degradation in liver cancer. Gastroenterology. 2006; 131: 1218-27.

[64] Singh RD, Schroeder AS, Scheffer L, et al. Prominin-2 expression increases protrusions, decreases caveolae and inhibits Cdc42 dependent fluid phase endocytosis. Biochem Biophys Res Commun. 2013; 434: 466-72.

[65] Takano S, Reichert M, Bakir B, et al. Prrx1 isoform switching regulates pancreatic cancer invasion and metastatic colonization. Genes Dev. 2016; 30: 233-47.

[66] Hirata H, Sugimachi K, Takahashi Y, et al. Downregulation of PRRX1 Confers Cancer Stem Cell-Like Properties and Predicts Poor Prognosis in Hepatocellular Carcinoma. Ann Surg Oncol. 2015; 22 Suppl 3: S1402-9.

[67] Takahashi Y, Sawada G, Kurashige J, et al. Paired related homoeobox 1, a new EMT inducer, is involved in metastasis and poor prognosis in colorectal cancer. Br J Cancer. 2013; 109: 307-11.

[68] Kashiwagi E, Shiota M, Yokomizo A, et al. Prostaglandin receptor EP3 mediates growth inhibitory effect of aspirin through androgen receptor and contributes to castration resistance in prostate cancer cells. Endocr Relat Cancer. 2013; 20: 431-41.

[69] Heidegger H, Dietlmeier S, Ye Y, et al. The Prostaglandin EP3 Receptor Is an Independent Negative Prognostic Factor for Cervical Cancer Patients. Int J Mol Sci. 2017; 18.

[70] Sui Y, Zheng X, Zhao D. Rab31 promoted hepatocellular carcinoma (HCC) progression via inhibition of cell apoptosis induced by PI3K/AKT/Bcl-2/BAX pathway. Tumour Biol. 2015; 36: 8661-70.

[71] Jin C, Rajabi H, Pitroda S, et al. Cooperative interaction between the MUC1-C oncoprotein and the Rab31 GTPase in estrogen receptor-positive breast cancer cells. PLoS One. 2012; 7: e39432.

[72] Kotzsch M, Dorn J, Doetzer K, et al. mRNA expression levels of the biological factors uPAR, uPAR-del4/5, and rab31, displaying prognostic value in breast cancer, are not clinically relevant in advanced ovarian cancer. Biol Chem. 2011; 392: 1047-51.

[73] Wang K, Wang B, Xing AY, Xu KS, Li GX, Yu ZH. Prognostic significance of SERPINE2 in gastric cancer and its biological function in SGC7901 cells. J Cancer Res Clin Oncol. 2015; 141: 805-12.

[74] An L, Lin Y, Yang T, Hua L. Exploring the interaction among EPHX1, GSTP1, SERPINE2, and TGFB1 contributing to the quantitative traits of chronic obstructive pulmonary disease in Chinese Han population. Hum Genomics. 2016; 10: 13.

[75] Sandsmark E, Andersen MK, Bofin AM, et al. SFRP4 gene expression is increased in aggressive prostate cancer. Sci Rep. 2017; 7: 14276.

[76] Deshmukh A, Kumar S, Arfuso F, Newsholme P, Dharmarajan A. Secreted Frizzled-related protein 4 (sFRP4) chemo-sensitizes cancer stem cells derived from human breast, prostate, and ovary tumor cell lines. Sci Rep. 2017; 7: 2256.

[77] Shi R, Yang Z, Liu W, Liu B, Xu Z, Zhang Z. Knockdown of Slit2 promotes growth and motility in gastric cancer cells via activation of AKT/beta-catenin. Oncol Rep. 2014; 31: 812-8.

[78] Qin F, Zhang H, Ma L, et al. Low Expression of Slit2 and Robo1 is Associated with Poor Prognosis and Brain-specific Metastasis of Breast Cancer Patients. Sci Rep. 2015; 5: 14430.

[79] Chang H, Dong T, Ma X, et al. Spondin 1 promotes metastatic progression through Fak and Src dependent pathway in human osteosarcoma. Biochem Biophys Res Commun. 2015; 464: 45-50.

[80] Liu CT, Zhu ST, Li P, Wang YJ, Zhang H, Zhang ST. SULF1 inhibits proliferation and invasion of esophageal squamous cell carcinoma cells by decreasing heparin-binding growth factor signaling. Dig Dis Sci. 2013; 58: 1256-63.

[81] Lai JP, Yu C, Moser CD, et al. SULF1 inhibits tumor growth and potentiates the effects of histone deacetylase inhibitors in hepatocellular carcinoma. Gastroenterology. 2006; 130: 2130-44.

[82] Adissu HA, McKerlie C, Di Grappa M, et al. Timp3 loss accelerates tumour invasion and increases prostate inflammation in a mouse model of prostate cancer. Prostate. 2015; 75: 1831-43.

[83] Han XG, Mo HM, Liu XQ, et al. TIMP3 Overexpression Improves the Sensitivity of Osteosarcoma to Cisplatin by Reducing IL-6 Production. Front Genet. 2018; 9: 135.

[84] Maleva Kostovska I, Jakimovska M, Popovska-Jankovic K, Kubelka-Sabit K, Karagjozov M, Plaseska-Karanfilska D. TIMP3 Promoter Methylation Represents an Epigenetic Marker of BRCA1ness Breast Cancer Tumours. Pathol Oncol Res. 2018.

[85] Zhang T, Shen X, Liu R, Zhu G, Bishop J, Xing M. Epigenetically upregulated WIPF1 plays a major role in BRAF V600E-promoted papillary thyroid cancer aggressiveness. Oncotarget. 2017; 8: 900-14.
