# Supplementary material for: Overexpressed MAGP1 Is Associated With a Poor Prognosis and Promotes Cell Migration and Invasion in Gastric Cancer
Source: Front Oncol. 2020 Jan 17;9:1544. doi: 10.3389/fonc.2019.01544 (PMC6978879; doi:10.3389/fonc.2019.01544)
Supplement: Table S2 — Association between MAGP1 expression and clinicopathological features of GC. [file Table_2.DOCX]

**Table S2 Association between MAGP1 expression and clinicopathological features of GC**

|  | No. patients | Low expression  (n = 34) | | High expression  (n = 40) | *P* value |
| --- | --- | --- | --- | --- | --- |
| Gender |  |  | |  |  |
| Male | 57 | 26 (45.6%) | | 31 (54.4%) | 0.916^a^ |
| Female | 17 | 8 (47.1%) | | 9 (52.9%) |  |
| Age |  |  | |  |  |
| ≤60 | 27 | 15 (55.6%) | | 12 (44.4%) | 0.209 ^a^ |
| >60 | 47 | 19 (40.4%) | | 28 (59.6%) |  |
| AJCC stage |  |  |  |  |  |
| I-II | 16 | 9 (56.3%) | | 7 (43.8%) | 0.350 ^a^ |
| III-IV | 58 | 25 (43.1%) | | 33 (56.9%) |  |
| T stage |  |  | |  |  |
| T1-2 | 7 | 3 (42.9%) | | 4 (57.1%) | 0.821^b^ |
| T3-4 | 67 | 31 (46.3%) | | 36 (53.7%) |  |
| N stage |  |  | |  |  |
| N0-1 | 33 | 21 (63.6%) | | 12 (36.4%) | 0.006 ^a^ |
| N2-3 | 41 | 13 (31.7%) | | 28 (68.3%) |  |
| M stage |  |  | |  |  |
| M0 | 62 | 32 (51.6%) | | 30 (48.4%) | 0.057 ^b^ |
| M1 | 12 | 2 (16.7%) | | 10 (83.3%) |  |
| Tumor size |  |  |  |  |  |
| ≤5cm | 45 | 22 (48.9%) | | 23 (51.1%) | 0.527 ^a^ |
| >5cm | 29 | 12 (41.4%) | | 17 (58.6%) |  |
| Differentiation |  |  |  |  |  |
| Well to moderate | 37 | 21 (56.8%) | | 16 (43.2%) | 0.062 ^a^ |
| Poor | 37 | 13 (35.1%) | | 24 (64.9%) |  |
| Borrmann classification |  |  | |  |  |
| I | 9 | 5 (55.6%) | | 4 (44.4%) | 0.199^b^ |
| II-III | 56 | 27 (48.2%) | | 29 (51.8%) |  |
| IV | 7 | 1 (14.3%) | | 6 (85.7%) |  |

a: chi-square test; b: Yates’ continuity corrected chi-square test.
