# Supplementary material for: Overexpressed MAGP1 Is Associated With a Poor Prognosis and Promotes Cell Migration and Invasion in Gastric Cancer
Source: Front Oncol. 2020 Jan 17;9:1544. doi: 10.3389/fonc.2019.01544 (PMC6978879; doi:10.3389/fonc.2019.01544)
Supplement: Table S4 — GO analysis of MAGP1 co-expressed genes in GC. [file Table_4.DOCX]

**Table S4 GO analysis of MAGP1 co-expressed genes in GC**

| **Category** | **GO Term** |  | **Number of Genes** | | | **Associated Genes(%)** | | **Term P Value** | | **Term FDR** | | **Associated Genes Found** | |  |
| --- | --- | --- | --- | --- | --- | --- | --- | --- | --- | --- | --- | --- | --- | --- |
| BP | GO:0030198~extracellular matrix organization | |  | | 54 | 7.407407 | | 4.18E-31 | | 7.4E-28 | | PXDN, PDGFB, POSTN, SERPINE1, TGFBI, LOX, COL11A1, LOXL1, COL10A1, MATN3, EGFL6, OLFML2B, BGN, COL1A2, VCAN, COL1A1, JAM3, MFAP5, TNC, LUM, ELN, COL3A1, ITGA11, ITGB5, COL2A1, DCN, COL9A2, CRISPLD2, ITGAV, COMP, COL6A3, COL6A2, COL6A1, FBN2, LAMB1, COL8A1, COL8A2, FN1, COL18A1, COL4A2, COL4A1, FBN1, CCDC80, NID2, SPARC, COL16A1, ECM2, COL5A2, COL5A1, EMILIN1, LAMA2, FBLN1, ITGA5, FBLN5 | |  |
| BP | GO:0007155~cell adhesion | |  | 68 | | | 9.327846 | | 2.74E-22 | | 4.84E-19 | | NUAK1, IGFBP7, L1CAM, BCAM, POSTN, KIAA1462, WISP1, SRPX, CTGF, FAP, TGFBI, COL12A1, SPON2, LOXL2, SPON1, EGFL6, PCDHB3, PCDHB4, PCDHB2, ACKR3, MFGE8, CERCAM, THY1, TNFAIP6, ANOS1, VCAN, TGFB1I1, COL1A1, MFAP4, ADAM12, TNC, PTK7, ITGA11, NINJ2, ITGB5, SPOCK1, CDH2, ITGBL1, ISLR, SORBS3, ITGAV, COMP, COL6A3, COL6A2, COL6A1, COL8A1, LAMB1, THBS2, THBS3, FN1, COL18A1, LRRN2, COL15A1, EFS, CD99, NID2, COL16A1, TPBG, COL5A1, EMILIN1, LAMA2, OMD, PKP1, ITGA5, NTM, MYH10, FEZ1, CDH11 | |
| BP | GO:0030574~collagen catabolic process | |  | 26 | | | 3.566529 | | 1.13E-19 | | 1.99E-16 | | ADAMTS14, COL3A1, COL2A1, MMP2, COL6A3, COL6A2, COL12A1, COL6A1, COL8A1, COL11A1, COL8A2, COL10A1, COL18A1, COL4A2, COL4A1, MRC2, MMP19, COL15A1, MMP14, COL5A2, COL5A1, MMP11, CTSK, COL1A2, COL1A1, ADAMTS2 | |
| BP | GO:0001501~skeletal system development | |  | 33 | | | 4.526749 | | 3.35E-17 | | 5.93E-14 | | FGFR1, AEBP1, NOG, PTH1R, COL3A1, SOX4, POSTN, COL2A1, TGFB2, COL9A2, COMP, NKX3-2, COL12A1, COL10A1, SH3PXD2B, MATN3, BMP1, EVC, SOX11, FBN1, IGF2, NPR3, COL5A2, PRELP, PTHLH, CDKN1C, DLX5, COL1A2, VCAN, COL1A1, CHRD, IGFBP4, CDH11 | |
| BP | GO:0030199~collagen fibril organization | |  | 18 | | | 2.469136 | | 9.7E-15 | | 1.71E-11 | | ADAMTS14, LUM, COL3A1, COL2A1, GREM1, COL5A2, SERPINH1, COL5A1, TGFB2, MMP11, SFRP2, COL1A2, COL12A1, COL1A1, LOX, LOXL2, ADAMTS2, COL11A1 | |
| BP | GO:0001649~osteoblast differentiation | |  | 23 | | | 3.155007 | | 2.76E-11 | | 4.87E-08 | | BMP4, NOG, TNC, MRC2, ITGA11, LEF1, GJA1, LRRC17, SNAI2, SNAI1, GLI1, SMO, DLX5, CREB3L1, COL6A1, VCAN, WNT11, COL1A1, TMEM119, IGFBP3, CHRD, RUNX2, TWIST1 | |
| BP | GO:0035987~endodermal cell differentiation | |  | 13 | | | 1.783265 | | 6.63E-11 | | 1.17E-07 | | INHBA, COL4A2, ITGA5, ITGAV, COL12A1, ITGB5, COL6A1, MMP14, COL8A1, LAMB1, COL11A1, MMP2, FN1 | |
| BP | GO:0001525~angiogenesis | |  | 32 | | | 4.389575 | | 2.47E-10 | | 4.37E-07 | | FGFR1, PGF, MMP2, TGFB2, SHB, HEY1, CTGF, FAP, ITGAV, TGFBI, SERPINE1, PLXND1, COL8A1, FGF1, COL8A2, FN1, COL18A1, RAMP2, COL4A2, MMP19, COL15A1, MFGE8, ACKR3, MMP14, ECM1, THY1, PRKD1, VEGFB, VEGFC, SRPX2, ITGA5, JAM3 | |
| BP | GO:0007275~multicellular organism development | |  | 52 | | | 7.133059 | | 2.8E-10 | | 4.96E-07 | | PDLIM7, PLXNA1, CCIN, GPR161, DZIP1, LTBP4, MOV10L1, RADIL, WNT2, OLFML3, LBH, HLX, CREB3L1, FGF1, TWIST2, PHC2, TMEFF1, EGFL6, MMP11, EYA4, SERPINF1, ROR2, WNT11, VCAN, SSC5D, TSHZ3, FGD1, ZNF516, MRAS, MFRP, PCOLCE, HIC1, SHISA2, HOXA4, ANGPTL2, MN1, THPO, BMP1, ST6GAL2, IGF2, FZD2, CSRP2, DKK2, DKK3, SALL4, PKP1, EBF4, SFRP2, MEOX1, SFRP4, ID3, CHRD | |
| BP | GO:0060325~face morphogenesis | |  | 11 | | | 1.508916 | | 7.27E-08 | | 0.000129 | | NOG, CRISPLD1, CRISPLD2, DLX5, TGFB3, LEF1, STRA6, COL1A1, MMP2, TGFB1, TGFB2 | |
| BP | GO:0030335~positive regulation of cell migration | |  | 25 | | | 3.429355 | | 9.17E-08 | | 0.000162 | | BMP4, COL18A1, PDGFB, SPHK1, LEF1, SNAI2, LRRC15, MMP14, SNAI1, TGFB1, GLI1, TNFAIP6, ITGA5, ITGAV, SEMA4C, PDGFRA, ROR2, PDGFRB, HAS2, PDGFC, WNT11, COL1A1, FGF1, LAMB1, PLAU | |
| BP | GO:0002576~platelet degranulation | |  | 18 | | | 2.469136 | | 2.37E-07 | | 0.000419 | | RARRES2, PDGFB, TGFB3, IGF2, SERPING1, PCDH7, SPARC, ECM1, TIMP3, TGFB1, FLNA, TIMP1, TGFB2, VEGFB, ISLR, VEGFC, SERPINE1, FN1 | |
| BP | GO:0001666~response to hypoxia | |  | 23 | | | 3.155007 | | 4.43E-07 | | 0.000783 | | NOX4, PLAT, RAMP2, CRYAB, PGF, TGFB3, POSTN, WTIP, MMP14, MMP2, ADORA1, TGFB1, AGTRAP, TGFB2, PKM, VEGFB, EDNRA, VEGFC, PLOD1, PLOD2, CYGB, LOXL2, PLAU | |
| BP | GO:0022617~extracellular matrix disassembly | |  | 15 | | | 2.057613 | | 6.9E-07 | | 0.00122 | | SH3PXD2B, BMP1, FBN1, ELN, MMP19, DCN, TIMP2, MMP14, MMP2, TIMP1, MMP11, CTSK, HTRA1, FBN2, FN1 | |
| BP | GO:0090090~negative regulation of canonical Wnt signaling pathway | |  | 22 | | | 3.017833 | | 7.15E-07 | | 0.001265 | | CTHRC1, LZTS2, NOG, HECW1, NKD2, IGFBP6, FZD1, LEF1, CDH2, SNAI2, GREM1, GLI1, DKK2, DKK3, DACT1, PRICKLE1, DACT3, SFRP2, SFRP4, ROR2, WNT11, IGFBP4 | |
| BP | GO:0001503~ossification | |  | 15 | | | 2.057613 | | 1.32E-06 | | 0.002335 | | BMP1, PDLIM7, FSTL3, MGP, LRRC17, IGF2, SPARC, ECM1, COL5A2, CTGF, TMEM119, RUNX2, COL11A1, TWIST1, CDH11 | |
| BP | GO:0032331~negative regulation of chondrocyte differentiation | |  | 8 | | | 1.097394 | | 1.34E-06 | | 0.002377 | | ADAMTS7, PTHLH, BMP4, EFEMP1, NKX3-2, ADAMTS12, GREM1, SNAI2 | |
| BP | GO:0045669~positive regulation of osteoblast differentiation | |  | 13 | | | 1.783265 | | 1.73E-06 | | 0.003064 | | PRKD1, BMP4, CTHRC1, CEBPB, PDLIM7, SFRP2, SOX11, FAM20C, CD276, GJA1, FBN2, TMEM119, RUNX2 | |
| BP | GO:0030178~negative regulation of Wnt signaling pathway | |  | 12 | | | 1.646091 | | 2.62E-06 | | 0.004627 | | DKK2, DKK3, BARX1, DACT1, NXN, SHISA2, DACT3, SFRP2, SFRP4, NFATC4, CITED1, HIC1 | |
| BP | GO:0007399~nervous system development | |  | 29 | | | 3.978052 | | 3.42E-06 | | 0.006051 | | NOG, NDN, FGF14, CTF1, GLIS2, NINJ2, SPOCK1, L1CAM, ST8SIA2, ADORA1, MDK, BARX1, GPSM1, DLG4, APBA2, ZNF423, GLRB, EFNB3, PCDHB3, PCDHB4, DPYSL4, PCDHB2, NUMBL, NBL1, INHBA, DOK5, HES4, DLX5, FEZ1 | |
| BP | GO:0007160~cell-matrix adhesion | |  | 15 | | | 2.057613 | | 5.62E-06 | | 0.009933 | | EPDR1, COL3A1, FERMT2, ITGA11, ITGB5, BCAM, NID2, ECM2, CTGF, ITGAV, FBLN5, SGCE, ADAMTS12, JAM3, THBS3 | |
| BP | GO:0071230~cellular response to amino acid stimulus | |  | 11 | | | 1.508916 | | 7.07E-06 | | 0.012512 | | COL4A1, CEBPB, COL3A1, COL1A2, COL6A1, PDGFC, COL1A1, ZEB1, COL16A1, MMP2, COL5A2 | |
| BP | GO:0051781~positive regulation of cell division | |  | 11 | | | 1.508916 | | 7.07E-06 | | 0.012512 | | VEGFB, VEGFC, PDGFB, PGF, TGFB3, IGF2, PDGFC, FGF1, MDK, TGFB1, TGFB2 | |
| BP | GO:0042060~wound healing | |  | 14 | | | 1.920439 | | 7.38E-06 | | 0.013058 | | NOG, TNC, COL3A1, TGFB3, PTK7, SPARC, DCN, TPM1, TIMP1, TGFB2, PDGFRA, PDGFRB, LOX, FN1 | |
| BP | GO:0060021~palate development | |  | 13 | | | 1.783265 | | 2.23E-05 | | 0.039444 | | TGFB3, PRRX1, LEF1, COL2A1, SNAI2, MSC, SNAI1, TGFB2, INHBA, BNC2, DLX5, WNT11, TWIST1 | |
| BP | GO:0030324~lung development | |  | 13 | | | 1.783265 | | 2.23E-05 | | 0.039444 | | WNT2, PDPN, CRISPLD2, CTGF, FSTL3, ZFPM2, STRA6, LOX, SPARC, FGF1, MMP14, ADAMTS2, GLI1 | |
| BP | GO:0007517~muscle organ development | |  | 14 | | | 1.920439 | | 2.42E-05 | | 0.04287 | | AEBP1, EVC, CRYAB, MRAS, CTF1, ITGA11, FHL3, LAMA2, TAGLN, COL6A3, SGCE, MKX, ID3, TWIST1 | |
| BP | GO:0001837~epithelial to mesenchymal transition | |  | 9 | | | 1.234568 | | 2.71E-05 | | 0.047939 | | NOG, LEF1, WNT11, LOXL3, SNAI2, LOXL2, SNAI1, TGFB1, TGFB2 | |
| MF | GO:0005201~extracellular matrix structural constituent | |  | 29 | | | 3.978052 | | 4.24E-23 | | 6.42E-20 | | PXDN, LUM, COL3A1, ELN, COL2A1, COMP, FBN2, LAMB1, COL11A1, COL8A2, MATN3, COL4A2, COL4A1, EFEMP2, FBN1, COL15A1, MGP, COL5A2, COL5A1, PRELP, FBLN1, BGN, FBLN2, COL1A2, ANOS1, VCAN, COL1A1, COL24A1, MFAP5 | |
| MF | GO:0048407~platelet-derived growth factor binding | |  | 10 | | | 1.371742 | | 5.5E-12 | | 8.33E-09 | | COL4A1, PDGFB, COL3A1, PDGFRA, COL1A2, PDGFRB, COL6A1, COL2A1, COL1A1, COL5A1 | |
| MF | GO:0005178~integrin binding | |  | 23 | | | 3.155007 | | 2.23E-11 | | 3.37E-08 | | EGFL6, LTBP4, TSPAN4, COL3A1, FBN1, MFGE8, ECM2, MMP14, COL16A1, TIMP2, COL5A1, THY1, FBLN1, WISP1, SFRP2, CTGF, ITGA5, FAP, FBLN5, TGFBI, FGF1, JAM3, FN1 | |
| MF | GO:0005518~collagen binding | |  | 18 | | | 2.469136 | | 2.54E-11 | | 3.84E-08 | | ASPN, PODN, PDGFB, LUM, MRC2, ITGA11, SPARC, DCN, NID2, LRRC15, SERPINH1, PCOLCE, P3H1, CTSK, COMP, TGFBI, ANTXR1, FN1 | |
| MF | GO:0005509~calcium ion binding | |  | 63 | | | 8.641975 | | 2.17E-10 | | 3.28E-07 | | ASPN, CLSTN2, LTBP2, LTBP3, LTBP4, FAM20C, SYT3, FSTL1, PCDHGA3, HMCN1, SLC24A3, SLC24A2, MATN3, SCUBE3, PCDHB5, EGFL6, PCDHB3, MMP19, PCDHB4, MGP, MMP17, PCDHB2, PCDH7, MMP14, MMP11, FKBP14, VCAN, FKBP10, PLA2G5, FKBP9, NKD2, FKBP7, EPDR1, MYL3, CD248, C1R, SPOCK1, C1S, CDH2, CALU, MYL9, MYL6B, COMP, PKD2, FBN2, THBS2, THBS3, GUCA1A, BMP1, MEX3B, EFEMP2, FBN1, EFEMP1, NID2, SPARC, NOTCH3, FBLN1, FBLN2, FBLN5, PLSCR3, SULF1, RCN3, CDH11 | |
| MF | GO:0008201~heparin binding | |  | 26 | | | 3.566529 | | 7.51E-10 | | 1.14E-06 | | FGFR1, LTBP2, FGF14, PGF, FSTL1, POSTN, MDK, PCOLCE, WISP1, CTGF, CRISPLD2, COMP, FGF1, THBS2, THBS3, FN1, BMP4, FBN1, CCDC80, ECM2, COL5A1, PRELP, VEGFB, ANOS1, CHRD, PLA2G5 | |
| MF | GO:0050840~extracellular matrix binding | |  | 11 | | | 1.508916 | | 1.2E-08 | | 1.82E-05 | | BGN, FBLN2, ITGAV, CD248, OLFML2B, TGFBI, ELN, DCN, SPARC, COL11A1, SSC5D | |
| MF | GO:0001968~fibronectin binding | |  | 10 | | | 1.371742 | | 1.91E-07 | | 0.000289 | | CTSK, FBLN1, SFRP2, CTGF, ITGAV, CCDC80, FSTL3, LRRC15, SSC5D, IGFBP3 | |
| MF | GO:0008083~growth factor activity | |  | 22 | | | 3.017833 | | 4.56E-07 | | 0.00069 | | BMP4, BMP1, PDGFB, FGF14, PGF, GDF6, EFEMP1, TGFB3, IGF2, MDK, TGFB1, CLEC11A, TGFB2, TIMP1, VEGFB, INHBA, VEGFC, CTGF, PDGFC, FGF1, THPO, NGF | |
| MF | GO:0016641~oxidoreductase activity, acting on the CH-NH2 group of donors, oxygen as acceptor | |  | 5 | | | 0.685871 | | 8.47E-06 | | 0.012825 | | LOXL4, LOXL3, LOX, LOXL2, LOXL1 | |
| MF | GO:0004222~metalloendopeptidase activity | |  | 16 | | | 2.194787 | | 1.48E-05 | | 0.022446 | | ADAMTS18, BMP1, ADAMTS14, ADAMTS16, MMP19, MMP17, MMP14, MMP2, MMP11, ADAMTS7, ADAMTS6, MMP23B, FAP, ADAMTS12, ADAM12, ADAMTS2 | |
| CC | GO:0005578~proteinaceous extracellular matrix | |  | 79 | | | 10.83676 | | 4.58E-48 | | 6.36E-45 | | ASPN, CTHRC1, PXDN, PODNL1, KERA, LTBP2, LTBP4, POSTN, MMP2, TGFB1, WNT2, WISP1, CILP2, CTGF, GPC6, TGFBI, LOX, SPON2, FGF1, COL11A1, SPON1, COL10A1, MATN3, OLFML2B, MMP19, MGP, MMP17, PRELP, MMP11, ADAMTS7, ADAMTS6, BGN, COL1A2, ANOS1, VCAN, WNT11, COL24A1, ADAMTS2, ADAMTS18, PODN, ADAMTS14, ADAMTS16, CD248, LUM, ELN, SPOCK1, TIMP2, TIMP3, CPZ, TIMP1, P3H1, COL9A2, CRISPLD2, COMP, COL6A3, COL6A2, ADAMTS12, FBN2, COL8A2, FN1, COL18A1, BMP4, BMP1, LGALS1, FBN1, EFEMP1, COL15A1, SPARC, ECM2, COL16A1, COL5A2, ECM1, COL5A1, EMILIN1, FBLN1, OMD, MMP23B, FBLN2, FBLN5 | |
| CC | GO:0031012~extracellular matrix | |  | 80 | | | 10.97394 | | 1.45E-45 | | 2.01E-42 | | ASPN, AEBP1, RARRES2, PXDN, LTBP2, PDGFB, LTBP3, IGFBP7, LTBP4, TGFB3, POSTN, MMP2, TGFB1, TGFB2, WNT2, PKM, HMCN1, HTRA1, SERPINE1, TGFBI, COL12A1, LOXL2, LOXL1, SPON1, MMP19, MGP, MMP17, MFGE8, MMP14, FLNA, PRELP, MMP11, BGN, SERPINF1, COL1A2, VCAN, TGFB1I1, COL1A1, MFAP4, SSC5D, TNC, LUM, VIM, COL3A1, COL2A1, DCN, TIMP2, TIMP3, PCOLCE, COMP, COL6A3, COL6A2, COL6A1, ADAMTS12, FBN2, COL8A1, LAMB1, THBS2, COL8A2, FN1, COL18A1, PLAT, COL4A2, COL4A1, LGALS1, FBN1, EFEMP1, LMCD1, COL15A1, NID2, COL5A2, ECM1, COL5A1, EMILIN1, LAMA2, FBLN1, MMP23B, FBLN2, SFRP2, FBLN5 | |
| CC | GO:0005576~extracellular region | |  | 155 | | | 21.262 | | 3.56E-29 | | 4.95E-26 | | RARRES2, NOG, KERA, PDGFB, LTBP3, PGF, FGF14, LTBP4, CAPZA2, GDF6, FSTL3, TGFB3, FSTL1, MMP2, TGFB1, TGFB2, WNT2, OLFML1, HTRA1, CTGF, SERPINE1, TGFBI, COL12A1, PDGFC, LOX, HTRA3, COL10A1, HHIPL1, TUB, MATN3, STC2, SCUBE3, OLFML2B, SERPING1, MFGE8, VASH2, PRELP, PTHLH, VEGFB, VEGFC, SERPINF1, PDGFRL, COL1A2, ANOS1, COL1A1, MFAP4, COL24A1, MFAP5, ADAMTS2, NGF, FGFR1, IL1R1, ELN, APOC2, CNPY4, POMC, TIMP2, MDK, TIMP3, TIMP1, CALU, FAM19A5, ITGBL1, LAMB1, OLFM2, PLTP, FN1, PLAT, BMP4, COL18A1, VSTM4, BMP1, EFEMP2, EFEMP1, COL16A1, ECM1, EMILIN1, NOTCH3, LAMA2, OMD, SFRP2, SFRP4, PLAU, CORIN, IGFBP7, ARSI, IGFBP6, HSD11B1L, SCT, LOXL3, GPX7, FGF1, COL11A1, LOXL1, MMP19, FLNA, MMP11, GRP, INHBA, CTSK, BGN, VCAN, WNT11, ADAM12, PLA2G5, ADAMTS14, EPDR1, TNC, LUM, OXT, CTF1, COL3A1, C1R, COL2A1, C1S, DCN, ISM1, ISLR, COL9A2, CRISPLD1, CRISPLD2, NPTX2, FNDC1, COMP, COL6A3, COL6A2, COL6A1, FBN2, COL8A1, FIBIN, THBS2, COL8A2, THBS3, THPO, COL4A2, COL4A1, IGLON5, FBN1, COL15A1, IGF2, NID2, SPARC, COL5A2, PLAC9, CLEC11A, COL5A1, DKK2, DKK3, C4ORF48, FBLN1, FAM180A, FBLN2, FBLN5, IGFBP3, IGFBP4 | |
| CC | GO:0005615~extracellular space | |  | 132 | | | 18.107 | | 2.72E-25 | | 3.78E-22 | | CTHRC1, NOG, PDGFB, LTBP2, KERA, PGF, GDF6, LTBP4, FAM20C, FSTL3, TGFB3, CNP, POSTN, FSTL1, MMP2, TGFB1, TGFB2, WNT2, HTRA1, CTGF, FAP, TGFBI, SERPINE1, COL12A1, PDGFC, LOX, SPON2, SPON1, STC2, CST2, SERPING1, CST1, MFGE8, PRELP, PTHLH, VEGFB, VEGFC, TNFAIP6, SERPINF1, CST4, COL1A2, ANOS1, COL1A1, SSC5D, PODN, APOC2, POMC, TIMP2, GREM1, TIMP3, SERPINH1, TIMP1, LAMB1, ANGPTL2, PLTP, FN1, COL18A1, BMP4, PLAT, BMP1, LGALS1, EFEMP1, LMCD1, ECM2, ECM1, OMD, SRPX2, SFRP2, SFRP4, CHRD, PLAU, PXDN, AEBP1, IGFBP7, IGFBP6, LRRC17, SCT, WISP1, GPC6, LOXL4, LOXL3, LOXL2, FGF1, LOXL1, VASN, EGFL6, ACTA2, GRP, CTSK, GNB2, CPXM1, SEMA4C, VCAN, WNT11, JAM3, CTSF, TNC, LUM, OXT, CTF1, COL3A1, COL2A1, SPOCK1, DCN, PCOLCE, CPZ, C1QTNF6, C1QTNF3, COMP, C1QTNF2, COL6A3, COL6A2, ENO2, THPO, TNFSF4, FBN1, COL15A1, AXL, NLGN2, IGF2, DPYSL3, SPARC, CLEC11A, DKK2, DKK3, NBL1, FBLN1, FBLN5, SULF1, IGFBP3, CMTM3, IGFBP4 | |
| CC | GO:0005788~endoplasmic reticulum lumen | |  | 37 | | | 5.075446 | | 7.23E-16 | | 1.08E-12 | | FKBP7, PDGFB, ARSI, COL3A1, COL2A1, SERPINH1, P3H1, KDELC1, COL9A2, P4HA3, COL6A3, COL6A2, COL12A1, COL6A1, PDGFC, GPX8, GPX7, COL8A1, COL11A1, COL8A2, SPON1, COL10A1, COL18A1, COL4A2, COL4A1, COL15A1, CERCAM, COL16A1, COL5A2, COL5A1, ADAMTS7, COL1A2, FKBP14, COL1A1, RCN3, FKBP10, COL24A1 | |
| CC | GO:0005581~collagen trimer | |  | 26 | | | 3.566529 | | 2.41E-15 | | 3.39E-12 | | CTHRC1, COL3A1, COL2A1, SERPINH1, PCOLCE, TIMP1, COL9A2, C1QTNF6, C1QTNF3, C1QTNF2, COL6A3, COL6A2, COL12A1, COL6A1, LOX, COL11A1, COL8A2, COL10A1, COL18A1, COL15A1, COL5A2, COL5A1, EMILIN1, COL1A2, COL1A1, COL24A1 | |
| CC | GO:0005604~basement membrane | |  | 23 | | | 3.155007 | | 6.62E-14 | | 9.18E-11 | | COL18A1, COL4A1, EGFL6, TNC, EFEMP2, FBN1, CCDC80, COL2A1, NID2, SPARC, TIMP3, COL5A1, TIMP1, LAMA2, FBLN1, HMCN1, SERPINF1, TGFBI, LOXL2, LAMB1, THBS2, LOXL1, COL8A2 | |
| CC | GO:0031093~platelet alpha granule lumen | |  | 13 | | | 1.783265 | | 6.35E-07 | | 0.000881 | | PDGFB, TGFB3, SERPING1, IGF2, SPARC, TGFB1, TIMP1, TGFB2, VEGFB, ISLR, VEGFC, SERPINE1, FN1 | |
| CC | GO:0005925~focal adhesion | |  | 36 | | | 4.938272 | | 1.55E-06 | | 0.002152 | | LIMS2, CNN3, PDLIM7, TSPAN4, TNC, VIM, FERMT2, FHL3, ITGA11, PTK7, PDLIM2, GJA1, ITGB5, L1CAM, TSPAN9, CDH2, SCARF2, SORBS3, FAP, ITGAV, NOX4, MRC2, FZD1, CD99, FZD2, CSRP2, MMP14, PALLD, FLNA, THY1, GNB2, ITGA5, SH3KBP1, PDGFRB, TGFB1I1, PLAU | |
| CC | GO:0009986~cell surface | |  | 43 | | | 5.898491 | | 5.97E-06 | | 0.00828 | | IL1R1, CLSTN2, PDGFB, CORIN, FERMT2, TGFB3, ITGB5, L1CAM, GREM1, TIMP2, TGFB1, SDC2, SRPX, ITGAV, FAP, PDGFC, RTN4RL2, FAM89B, PLAT, VASN, RAMP2, HYAL2, TNFSF4, SCUBE3, CRYAB, LGALS1, FZD1, AXL, NLGN2, ACKR3, SPARC, MXRA8, TPBG, ADAMTS7, BGN, SRPX2, ITGA5, SFRP4, SULF1, PDGFRB, ANTXR1, ADGRA2, PLAU | |
