# Supplementary material for: Overexpressed MAGP1 Is Associated With a Poor Prognosis and Promotes Cell Migration and Invasion in Gastric Cancer
Source: Front Oncol. 2020 Jan 17;9:1544. doi: 10.3389/fonc.2019.01544 (PMC6978879; doi:10.3389/fonc.2019.01544)
Supplement: Table S5 — KEGG pathway analysis of MAGP1 co-expressed genes in GC. [file Table_5.DOCX]

**Table S5 KEGG pathway analysis of MAGP1 co-expressed genes in GC**

| **GO Term** | **Number of genes** | **Associated genes(%)** | **Term P Value** | **Term FDR** | **Associated Genes Found** |
| --- | --- | --- | --- | --- | --- |
| hsa04512:ECM-receptor interaction | 25 | 3.429355 | 7.99E-16 | 9.77E-13 | TNC, COL3A1, ITGA11, ITGB5, COL2A1, COMP, ITGAV, COL6A3, COL6A2, COL6A1, SV2A, LAMB1, COL11A1, THBS2, THBS3, FN1, COL4A2, COL4A1, COL5A2, COL5A1, LAMA2, ITGA5, COL1A2, COL1A1, COL24A1 |
| hsa04510:Focal adhesion | 34 | 4.663923 | 7.39E-14 | 9.30E-11 | PDGFB, PGF, TNC, COL3A1, ITGA11, ITGB5, COL2A1, MYL9, ITGAV, COMP, COL6A3, COL6A2, COL6A1, PDGFC, LAMB1, COL11A1, THBS2, THBS3, SHC4, FN1, COL4A2, COL4A1, COL5A2, FLNA, COL5A1, LAMA2, VEGFB, VEGFC, ITGA5, COL1A2, PDGFRA, PDGFRB, COL1A1, COL24A1 |
| hsa04974:Protein digestion and absorption | 19 | 2.60631 | 7.95E-10 | 9.99E-07 | COL18A1, COL4A2, COL4A1, COL3A1, ELN, COL15A1, COL2A1, COL5A2, COL5A1, COL9A2, COL6A3, COL1A2, COL6A2, COL12A1, COL6A1, COL1A1, COL24A1, COL11A1, COL10A1 |
| hsa04151:PI3K-Akt signaling pathway | 37 | 5.075446 | 1.87E-09 | 2.35E-06 | FGFR1, PDGFB, FGF14, PGF, TNC, COL3A1, ITGA11, ITGB5, COL2A1, ITGAV, COMP, COL6A3, COL6A2, COL6A1, CREB3L1, PDGFC, LAMB1, FGF1, THBS2, COL11A1, THBS3, FN1, COL4A2, COL4A1, COL5A2, COL5A1, LAMA2, VEGFB, VEGFC, GNB2, ITGA5, COL1A2, PDGFRA, PDGFRB, COL1A1, COL24A1, NGF |
| hsa05205:Proteoglycans in cancer | 24 | 3.292181 | 3.56E-07 | 4.47E-04 | FGFR1, MRAS, LUM, FZD1, ITGB5, IGF2, DCN, FZD2, MMP2, TIMP3, SDC2, TGFB1, FLNA, TGFB2, WNT2, SMO, ITGA5, ITGAV, HSPB2, WNT11, PLAU, TWIST2, FN1, TWIST1 |
| hsa05146:Amoebiasis | 17 | 2.331962 | 6.17E-07 | 7.76E-04 | COL4A2, IL1R1, COL4A1, COL3A1, TGFB3, COL2A1, COL5A2, TGFB1, COL5A1, TGFB2, LAMA2, COL1A2, COL1A1, LAMB1, COL24A1, COL11A1, FN1 |
| hsa04390:Hippo signaling pathway | 20 | 2.743484 | 9.80E-07 | 0.001232 | BMP4, GDF6, FZD1, TGFB3, LEF1, TEAD2, WTIP, FZD2, SNAI2, TGFB1, TCF7L1, TGFB2, WNT2, FRMD6, CTGF, SERPINE1, DLG4, WNT11, PARD6G, FGF1 |
| hsa05200:Pathways in cancer | 33 | 4.526749 | 4.74E-06 | 0.005966 | TRAF1, CKS1B, FGFR1, PDGFB, FGF14, PGF, TGFB3, MMP2, TCF7L1, TGFB1, TGFB2, GLI1, EDNRA, WNT2, ITGAV, FGF1, LAMB1, FN1, BMP4, COL4A2, PTGER3, COL4A1, FZD1, LEF1, FZD2, LAMA2, VEGFB, SMO, VEGFC, GNB2, PDGFRA, PDGFRB, WNT11 |
| hsa04350:TGF-beta signaling pathway | 13 | 1.783265 | 2.88E-05 | 0.036206 | BMP4, NBL1, INHBA, NOG, GDF6, TGFB3, SMURF2, TGIF2, ID3, DCN, CHRD, TGFB1, TGFB2 |
| hsa05217:Basal cell carcinoma | 9 | 1.234568 | 4.93E-04 | 0.617569 | WNT2, BMP4, SMO, FZD1, LEF1, WNT11, FZD2, TCF7L1, GLI1 |
| hsa05410:Hypertrophic cardiomyopathy (HCM) | 10 | 1.371742 | 0.001429221 | 1.782397 | MYL3, ITGA5, ITGAV, TGFB3, ITGA11, ITGB5, TPM2, TPM1, TGFB1, TGFB2 |
| hsa05414:Dilated cardiomyopathy | 10 | 1.371742 | 0.002409259 | 2.987662 | MYL3, ITGA5, ITGAV, TGFB3, ITGA11, ITGB5, TPM2, TPM1, TGFB1, TGFB2 |
| hsa04540:Gap junction | 10 | 1.371742 | 0.003316474 | 4.091218 | PDGFB, PDGFRA, PDGFRB, GUCY1A3, GJA1, TUBB6, GUCY1B3, PDGFC, TUBA1A, TUBB3 |
| hsa05144:Malaria | 7 | 0.960219 | 0.006608998 | 7.999976 | COMP, TGFB3, THBS2, TGFB1, THBS3, SDC2, TGFB2 |
| hsa05412:Arrhythmogenic right ventricular cardiomyopathy (ARVC) | 8 | 1.097394 | 0.008252774 | 9.896027 | ITGA5, ITGAV, ITGA11, ITGB5, LEF1, GJA1, CDH2, TCF7L1 |
| hsa04310:Wnt signaling pathway | 12 | 1.646091 | 0.008354145 | 10.01177 | DKK2, WNT2, NKD2, PRICKLE1, SFRP2, SFRP4, FZD1, LEF1, NFATC4, WNT11, FZD2, TCF7L1 |
| hsa04014:Ras signaling pathway | 16 | 2.194787 | 0.011929424 | 14.00746 | FGFR1, PDGFB, PGF, FGF14, MRAS, VEGFB, VEGFC, RASGRF2, GNB2, PDGFRA, PDGFRB, PDGFC, FGF1, PLA2G5, NGF, SHC4 |
| hsa04810:Regulation of actin cytoskeleton | 15 | 2.057613 | 0.014347659 | 16.61676 | FGFR1, FGD1, PDGFB, FGF14, MRAS, ITGA11, ITGB5, MYL9, ITGA5, ITGAV, PDGFRA, PDGFRB, PDGFC, FGF1, FN1 |
| hsa04270:Vascular smooth muscle contraction | 10 | 1.371742 | 0.020198713 | 22.63154 | EDNRA, RAMP2, ACTA2, MYL6B, CALD1, GUCY1A3, GUCY1B3, PLA2G5, PPP1R14A, MYL9 |
| hsa04514:Cell adhesion molecules (CAMs) | 11 | 1.508916 | 0.025679742 | 27.90107 | ITGAV, CD276, NTNG2, CD99, NLGN2, VCAN, L1CAM, CLDN11, CDH2, JAM3, SDC2 |
| hsa05222:Small cell lung cancer | 8 | 1.097394 | 0.02778242 | 29.83338 | LAMA2, TRAF1, CKS1B, COL4A2, COL4A1, ITGAV, LAMB1, FN1 |
| hsa00532:Glycosaminoglycan biosynthesis - chondroitin sulfate / dermatan sulfate | 4 | 0.548697 | 0.030504029 | 32.2637 | CHSY3, CHST12, CHST14, CHST3 |
| hsa04015:Rap1 signaling pathway | 14 | 1.920439 | 0.030790868 | 32.51528 | VEGFB, PRKD1, VEGFC, FGFR1, PDGFB, FGF14, PGF, MRAS, PDGFRA, PDGFRB, PDGFC, PARD6G, FGF1, NGF |
| hsa05215:Prostate cancer | 8 | 1.097394 | 0.032772664 | 34.23006 | FGFR1, PDGFB, PDGFRA, PDGFRB, CREB3L1, LEF1, PDGFC, TCF7L1 |
| hsa04145:Phagosome | 11 | 1.508916 | 0.035637337 | 36.63796 | ITGA5, ITGAV, COMP, MRC2, ITGB5, TUBB6, C1R, TUBA1A, THBS2, THBS3, TUBB3 |
| hsa05218:Melanoma | 7 | 0.960219 | 0.036148184 | 37.05872 | FGFR1, PDGFB, FGF14, PDGFRA, PDGFRB, PDGFC, FGF1 |
| hsa04611:Platelet activation | 10 | 1.371742 | 0.036675996 | 37.49076 | COL3A1, COL1A2, GUCY1A3, COL2A1, GUCY1B3, COL1A1, COL24A1, COL11A1, COL5A2, COL5A1 |
