# Supplementary figures and images for: Overexpressed MAGP1 Is Associated With a Poor Prognosis and Promotes Cell Migration and Invasion in Gastric Cancer
Source: Front Oncol. 2020 Jan 17;9:1544. doi: 10.3389/fonc.2019.01544 (PMC6978879; doi:10.3389/fonc.2019.01544)

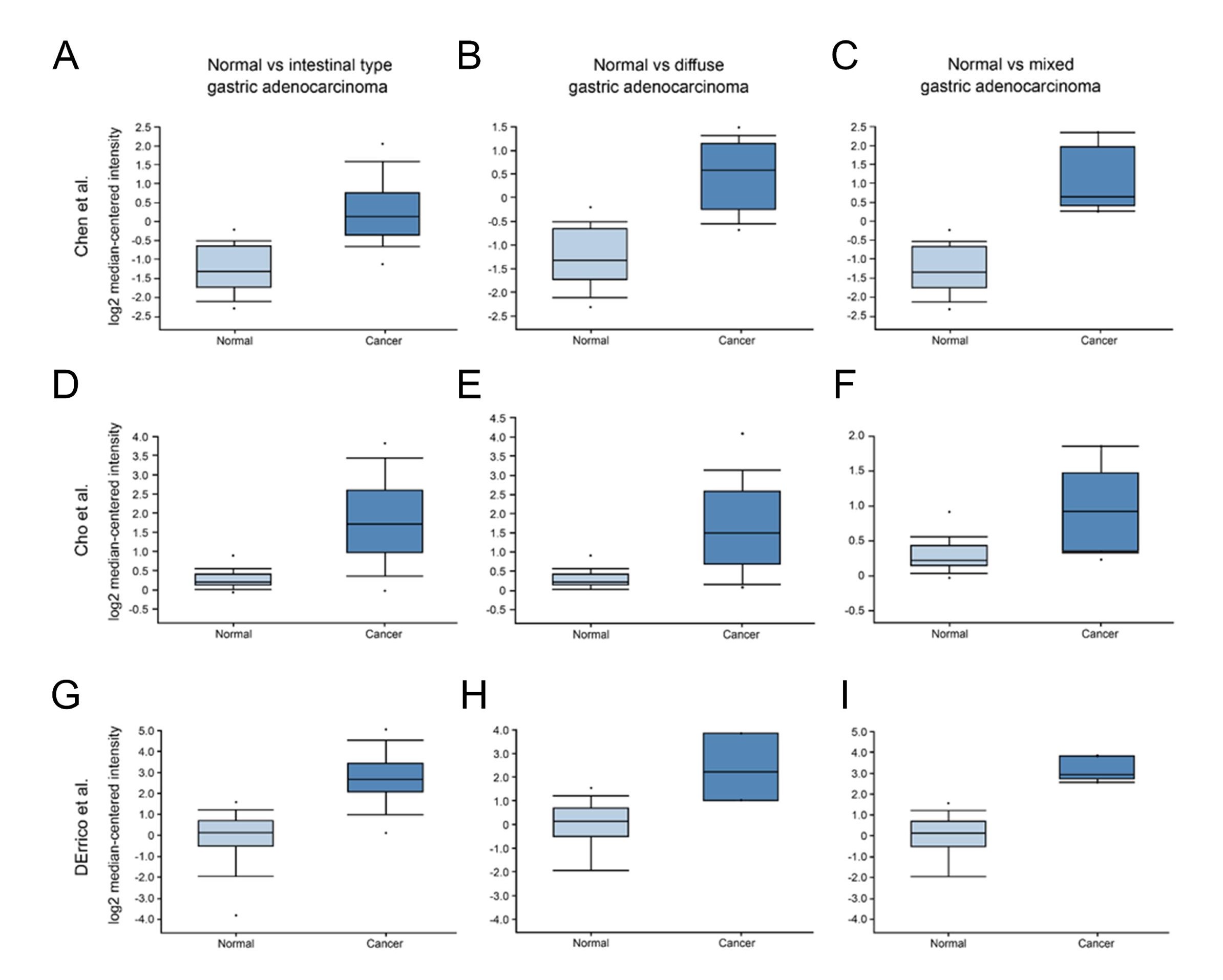

Supplement: Figure S1 — MAGP1 mRNA expression in different gastric Lauren classifications. Analysis of MAGP1 mRNA expression in Chen et al. (A–C), Cho et al. (D–F), and Derrico et al. (G–I) gastric datasets. Boxes, 25th−75th percentile; whisker, 10th and 90th percentile; points, minimum and maximum. All data was achieved from Oncomine database. [file Image_1.JPEG]

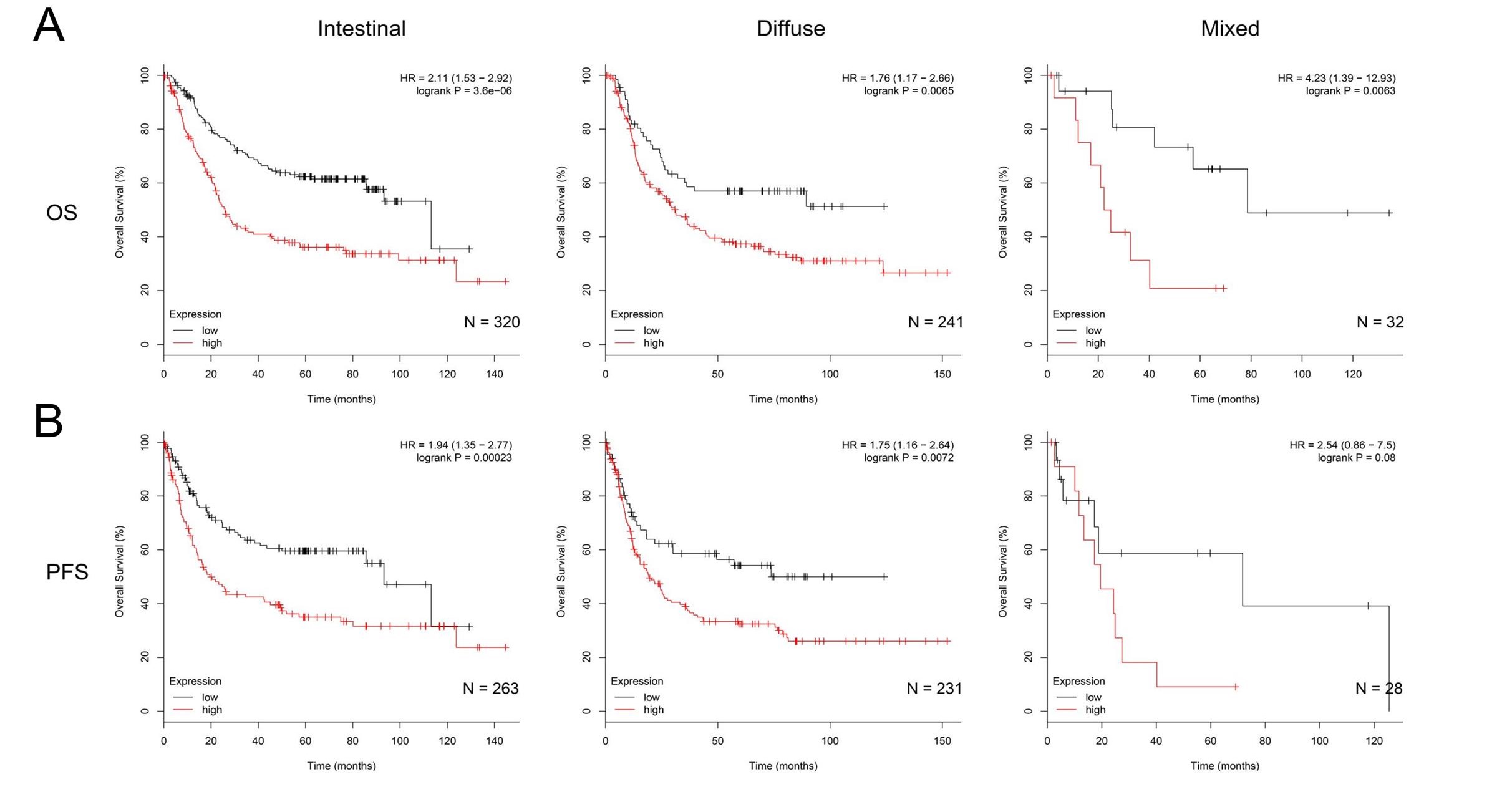

Supplement: Figure S2 — The association between mfap2 mRNA expression and prognosis of gastric cancer with different Lauren classifications using K–M plotter data. (A) Overall survival was showed between high and low expression group of mfap2 in intestinal, diffuse, and mixed Lauren classification. (B) Progress survival was showed between high and low expression group of mfap2 in intestinal, diffuse, and mixed Lauren classification. [file Image_2.JPEG]

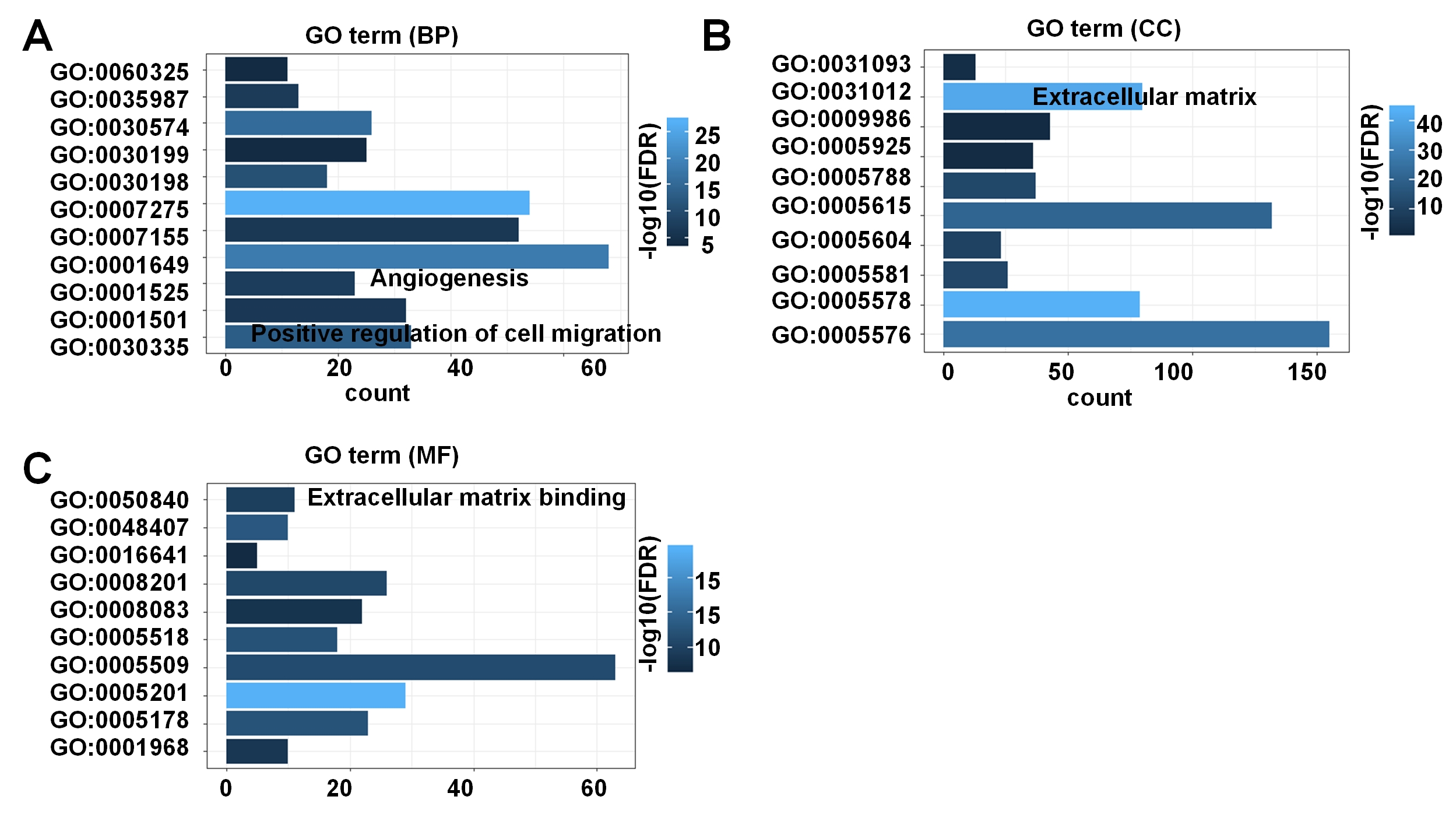

Supplement: Figure S3 — GO analysis of MAGP1 co-expressed genes in GC. (A) Biological processes (BPs); (B) Cellular components (CCs); (C) Molecular factors (MFs). GO, gene ontology. [file Image_3.JPEG]

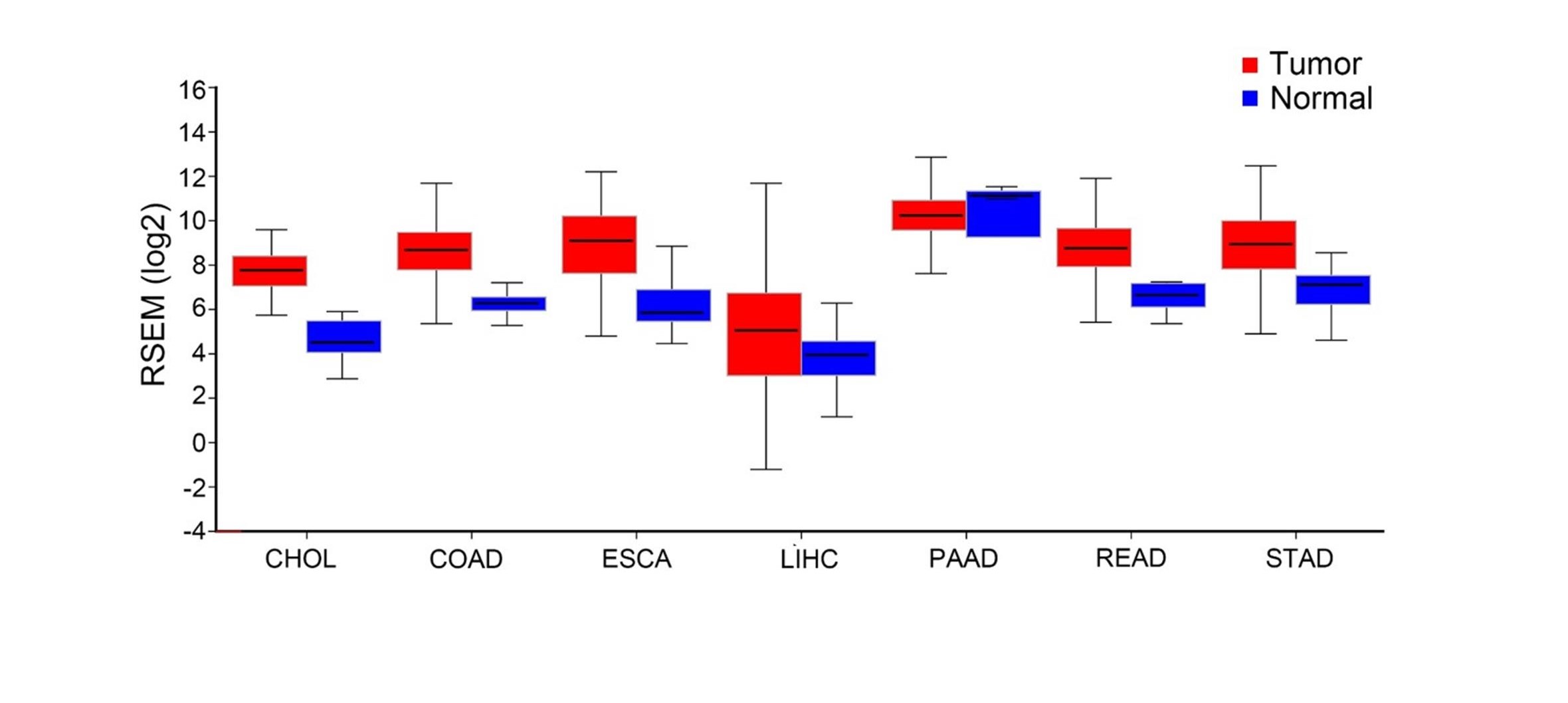

Supplement: Figure S4 — The MAGP1 mRNA expression in digestive system tumors using Firehose. Red color represented tumors and blue color represented corresponding normal tissues. RSEM, RNASeq by expectation maximization. CHOL, cholangiocarcinoma; COAD, colon adenocarcinoma; ESCA, esophageal carcinoma; LIHC, liver hepatocellular carcinoma; PAAD, pancreatic adenocarcinoma; READ, rectum adenocarcinoma; STAD, stomach adenocarcinoma. [file Image_4.JPEG]
